# Interphase-arrested *Drosophila* embryos activate zygotic gene expression and initiate mid-blastula transition events at a low nuclear-cytoplasmic ratio

**DOI:** 10.1371/journal.pbio.3000891

**Published:** 2020-10-22

**Authors:** Isaac J. T. Strong, Xiaoyun Lei, Fang Chen, Kai Yuan, Patrick H. O’Farrell

**Affiliations:** 1 Hunan Key Laboratory of Molecular Precision Medicine, Department of Oncology, Xiangya Hospital, Central South University, Changsha, Hunan, China; 2 Hunan Key Laboratory of Medical Genetics, School of Life Sciences, Central South University, Changsha, Hunan, China; 3 Department of Biochemistry and Biophysics, University of California San Francisco, San Francisco, California, United States of America; The Institute of Cancer Research, UNITED KINGDOM

## Abstract

Externally deposited eggs begin development with an immense cytoplasm and a single overwhelmed nucleus. Rapid mitotic cycles restore normality as the ratio of nuclei to cytoplasm (N/C) increases. A threshold N/C has been widely proposed to activate zygotic genome transcription and onset of morphogenesis at the mid-blastula transition (MBT). To test whether a threshold N/C is required for these events, we blocked N/C increase by down-regulating cyclin/Cdk1 to arrest early cell cycles in *Drosophila*. Embryos that were arrested two cell cycles prior to the normal MBT activated widespread transcription of the zygotic genome including genes previously described as N/C dependent. Zygotic transcription of these genes largely retained features of their regulation in space and time. Furthermore, zygotically regulated post-MBT events such as cellularization and gastrulation movements occurred in these cell cycle–arrested embryos. These results are not compatible with models suggesting that these MBT events are directly coupled to N/C. Cyclin/Cdk1 activity normally declines in tight association with increasing N/C and is regulated by N/C. By experimentally promoting the decrease in cyclin/Cdk1, we uncoupled MBT from N/C increase, arguing that N/C-guided down-regulation of cyclin/Cdk1 is sufficient for genome activation and MBT.

## Abstract 2

Externally deposited eggs begin development with an immense cytoplasm and a single overwhelmed nucleus. This study shows that experimentally arresting the cell cycle bypasses the requirement of a threshold nuclei-to-cytoplasm (N/C) ratio for activation of the zygotic genome and other mid-blastula transition events in *Drosophila* early embryos.

## Introduction

Unlike mammalian eggs, whose growth and development is fostered in a nutrient-rich environment, the ancestral and common program of development requires eggs that provide for external development into free-living organisms [1, 2]. These autonomously developing eggs start with a massive cytoplasmic volume. The egg, with its single diploid nucleus, does not have the capacity to rapidly adjust the population of transcripts in its huge cytoplasm [2, 3]. Accordingly, the nucleus forgoes its usual commanding role in regulation, and a maternal program runs early development independent of zygotic transcription. This maternal program is largely dedicated to rapid cell cycles that amplify the zygotic genome [4, 5]. When the nuclei achieve the capacity to take command, the biology of the early embryo is transformed at the mid-blastula transition (MBT). At this stage, the cell cycle slows, the maternal posttranscriptional program is shut down, zygotic transcription is up-regulated, cells start to take on distinct fates, and morphogenesis begins [4–15].

Maternally deposited proteins and RNAs guide *Drosophila* embryos through rapid early syncytial cell cycles that slow incrementally as they approach mitosis 13 [16–19]. Though a number of important genes are transcribed during the prelude to the MBT, they are not needed until later [18, 20, 21]. Completion of mitosis 13 and the onset of cycle 14 mark the beginning of the MBT [4]. At this time, the cell cycle is abruptly extended and transcription is activated more widely [4, 16–19]. Other events within interphase of cell cycle 14 highlight the transition. Many maternal gene products are eliminated, several histone modifications make their first appearance, and the 3D chromatin architecture is formed [16, 20, 22–30]. Zygotic transcriptional cascades direct key events of post-MBT embryos; these events include the cellularization of the approximately 6,000 cortical nuclei produced during the early 13 rapid syncytial divisions, patterning of the embryo, and the onset of gastrulation movements that transform embryonic shape [4, 31].

Here we are concerned with the timing and coordination of these remarkable events. Although more than one factor might contribute to the timing of the MBT, manipulation of the DNA content and cytoplasmic volume of early embryos has led to the conclusion that the ratio of nuclei to cytoplasm (N/C) makes a major contribution [17, 32]. In *Drosophila*, the key manipulations of N/C have been genetic [17, 18]. Haploid embryos, which take one extra cell cycle to achieve the same N/C as a diploid embryo, undergo an additional rapid and synchronous mitotic cycle before gastrulation. This work in *Drosophila* is consistent with the prevailing view that the exponential increase in nuclei during the early rapid embryonic cycles contributes to timing of the MBT.

How the N/C contributes to the MBT remains debated. A common model posits that N/C directly controls the activation of zygotic transcription, which in turn relays the signal to other downstream MBT events. This model is supported by several lines of evidence collected from studies using haploid embryos. First, the zygotic expression of a group of genes showed N/C dependency. When the transcriptomes of the MBT-delayed haploid embryos were compared to that of the diploid embryos using microarray, 88 genes were identified as “N/C dependent” because their expression was delayed by one cell cycle in the haploid embryos [33]. Second, N/C seemed to control the accessibility of many promoters [34], which coincided with the large-scale recruitment of RNA polymerase II (Pol II) to genes prior to MBT. The recruited Pol II could then lengthen the cell cycle by triggering the DNA replication checkpoint [28]. Third, N/C modulated the degradation of Cdc25^Twine^, a phosphatase that activates cyclin/Cdk1 by removing its inhibitory phosphorylation and thus regulates cell cycle remodeling at the MBT [35–37]. Lastly, a more recent preprint suggested that N/C could directly influence the kinetics of transcription activation as well as the probability of transcription initiation for certain genes [38].

However, the original analysis of haploid *Drosophila* embryos that showed miscoordination of developmental events was interpreted in a different way [17, 18, 39]. The commonly quoted result from this analysis of haploid embryos is that cellularization, a post-MBT event that requires zygotic transcription, did not complete until cycle 15 instead of the normal cycle 14. This incomplete description ignores the finding that cellularization initiated at roughly the normal time, often beginning in the cycle 14 as in diploid embryos, but the prolonged process of cellularization was interrupted by a premature mitosis 14 and, hence, was only completed in the next cell cycle. From this and other findings, the authors concluded that the temporal miscoordination in haploid embryos was due to a defect in the slowing of the cell cycle [17], and through an analysis of transcription they further concluded “transcriptional activation is not directly controlled by the nucleocytoplasmic ratio” [17, 18].

Activation of zygotic transcription and remodeling of the cell cycle in the early embryos are often tightly entangled, and it has been difficult to distinguish among different possible hierarchies of regulatory coupling. It could be that transcriptional activation of N/C-dependent genes triggers the slowing of the cell cycle, or that the rising N/C slows the cell cycle to trigger transcriptional activation, or N/C independently but reliably triggers both events.

Here we use a different type of experiment to test whether MBT events, especially zygotic genome activation (ZGA), require a threshold N/C, and we provide an indication that N/C indirectly regulates ZGA through its impact on the activity of cyclin/Cdk1. We knocked down cyclin/Cdk1 to block the early cell cycles [40]. These cell cycle–arrested embryos never reached an N/C characteristic of the MBT. We found that arrested embryos still exhibited numerous post-MBT events including complex morphogenesis and patterning, arguing that a threshold N/C is not the direct trigger of these events. RNA sequencing (RNA-seq) analyses of the cell cycle–arrested embryos showed large-scale activation of zygotic transcription including previously defined “N/C-dependent” genes. Thus, our experimental manipulation uncoupled MBT events from the normal increase in N/C. Based on what happens in undisturbed embryos, we suggest that down-regulation of cyclin/Cdk1 normally triggers the MBT and ZGA.

## Results

### Cellularization after blocking nuclear cycles prior to the MBT

Previously, we showed that injection of double-stranded RNA (dsRNA) complementary to all three of the *Drosophila* mitotic cyclins, cyclin A, cyclin B, and cyclin B3, blocks early embryonic cell cycles in interphase [35, 40–42]. While our early experiments focused on the cell cycle roles of cyclins, we noted a surprising lack of an effect of cell cycle arrest on MBT events. This was particularly evident in embryos injected with RNA interference (RNAi) at one pole that gave rise to embryos with three zones arrested in cycles 12 through 14, all of which cellularized at the same time as if local N/C had little impact [37, 40]. The absence of local influence of N/C stands in contrast to the observations of Lu and colleagues [33], suggesting that our interruption of cell cycle progress was bypassing N/C inputs. In the present study, we injected dsRNA at three points along the A/P axis of embryos to induce uniform arrest of the cell cycle and explored the effects on MBT events—in particular, zygotic transcription.

**Fig 1:**
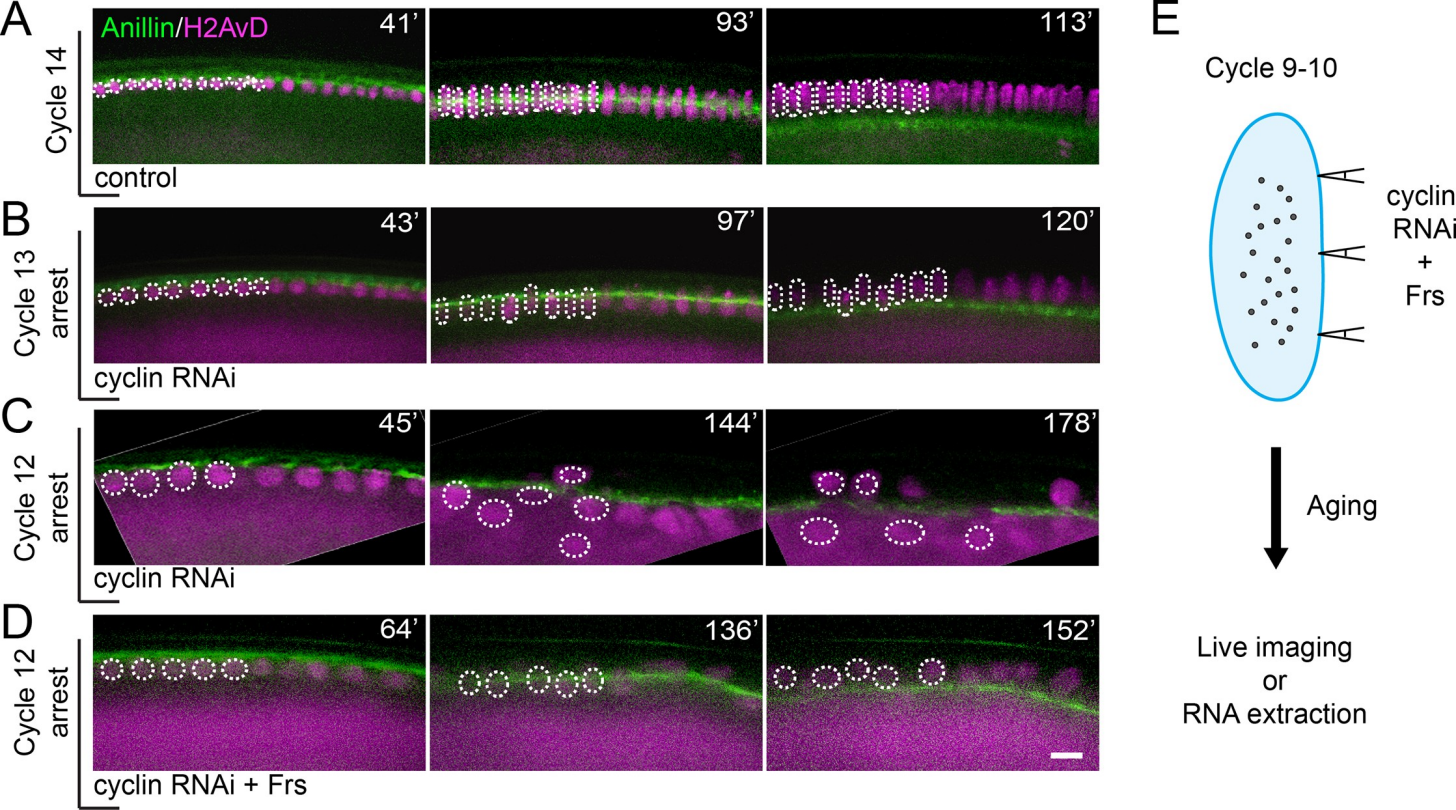
Combination of cyclin RNAi with Cdk1 inhibitor Frs allows cellularization in embryos arrested in cycle 12. **(A-D)** Visualization of cellularization in control and cell cycle–arrested embryos in transverse section. The upper boundary of YFP-Anillin (green) (left most) shows the surface of the embryo, and the bright descending front marks the advancing cellularization furrows. Histone H2AvD-mRFP (purple) shows nuclei and a subcortical cytoplasmic pool. Ingression is orderly and nuclei stable in the control (A) and in embryos arrested in nuclear cycle 13 (B). Furrows still ingress in embryos arrested in nuclear cycle 12 but the nuclei are thrown into disarray (C). A combined injection of cyclin RNAi and Frs protein, a Cdk1 inhibitor, allows more orderly cellularization in embryos arrested as early as cycle 12 (D). Several nuclei are highlighted by dotted circles for ease of following their positions relative to cellularization furrows (green). The numbers at the top-right corner of each image indicate elapsed time starting from the beginning of cycle 12 (in minutes). Note that the subcortical pool of Histone H2AvD-mRFP is very bright in arrested embryos, presumably reflecting continued translation without further recruitment to DNA when S phases are blocked (S1A Fig). Bar: 15 μm. **(E)** The combined injection scheme used to induce early embryonic cell cycle arrest in this study. The effectiveness of the arrest is further detailed in S1 Fig. Frs, Frühstart; mRFP, monomeric red fluorescent protein; RNAi, RNA interference; YFP, yellow fluorescent protein.

Embryos arrested in cycle 13 underwent cellularization (Fig 1A and 1B), a morphological hallmark of MBT that requires zygotic transcription [22, 26]. This suggests that cellularization, and the associated zygotic transcription, does not need progression to cell cycle 14. However, early embryonic cycles lack a G1 phase, and the knockdown of the three mitotic cyclins allows completion of S phase before arrest in an artificial G2-like state [35, 42] (S1A Fig). Hence, arrested cycle 13 embryos have a DNA content equivalent to normal embryos as they enter cycle 14 prior to S phase 14. To achieve a cell cycle arrest truly below the MBT threshold of DNA, we need to arrest embryos in cycle 12 or earlier.

Slightly earlier injection of cyclin dsRNA arrested the nuclei in cycle 12; however, as previously reported, the centrosomes continued to replicate (S1B Fig, middle panel) [40]. Additionally, about the time we would have expected cellularization, such arrested embryos exhibited anomalous chaotic cytoplasmic movements and disruption of the cortical nuclear arrangement (e.g., Fig 1C). Frühstart (Frs) is a Cdk1/Cdk2 inhibitor normally expressed in early cycle 14. Its introduction in pre–cycle 14 embryos was shown to transiently arrest the cell cycle [43, 44]. We found that injection of cyclin dsRNA combined with Frs protein (Fig 1E) resulted in a more complete early embryonic cell cycle arrest with an arrested centrosome duplication cycle (S1B Fig). Embryos arrested in cycle 12 by this protocol maintained an organized cortex and cellularized without major disruption (Fig 1D). Thus, cellularization does not require a cycle 14 DNA content, at least under our conditions of cyclin/Cdk inhibition.

### Gastrulation and embryonic patterning in cell cycle–arrested embryos

**Fig 2:**
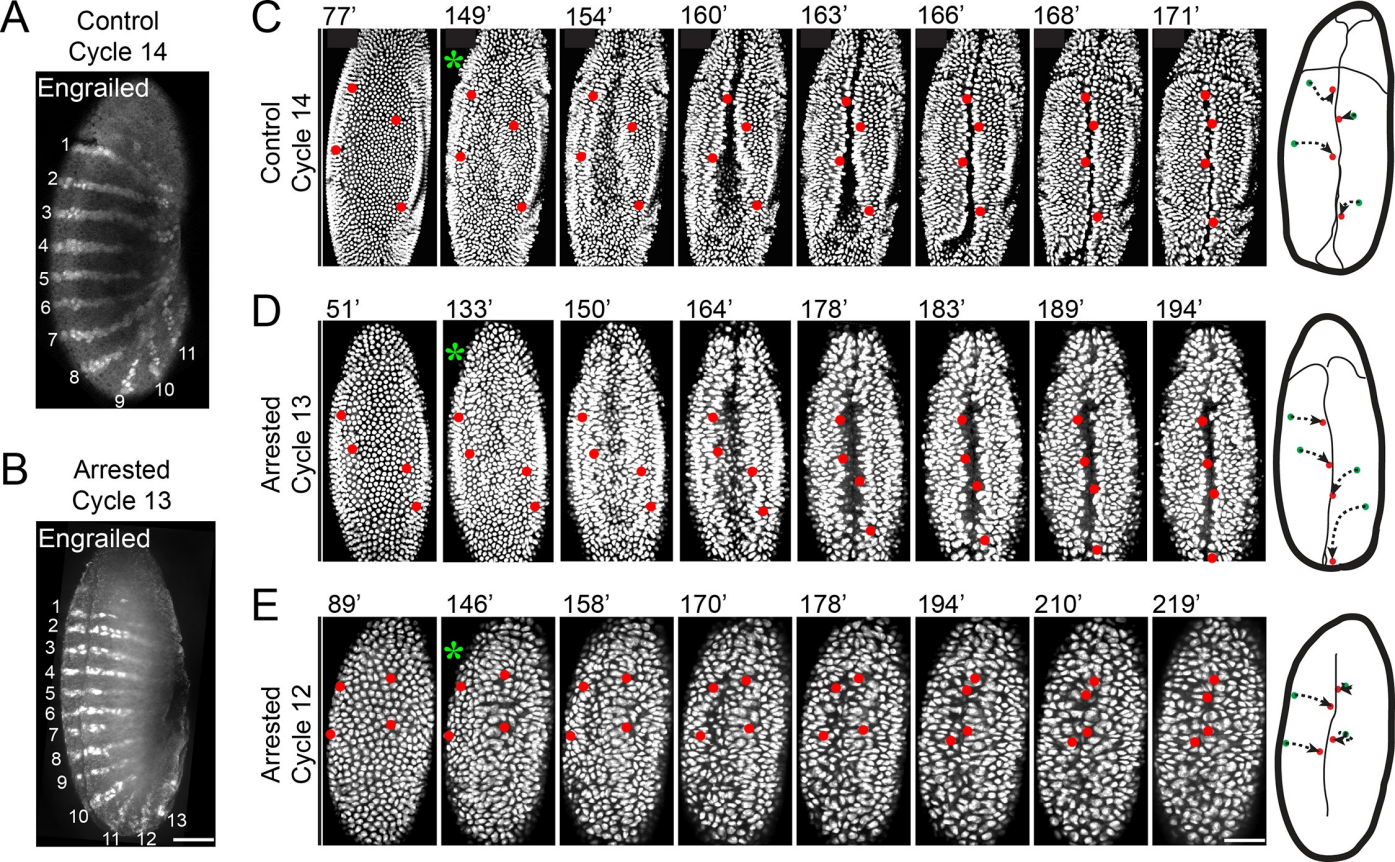
Embryonic patterning and gastrulation in cell cycle–arrested embryos. **(A-B)** Establishment of the Engrailed protein expression pattern with fewer nuclei. Embryos arrested in cycle 13 (B) display Engrailed stripes comparable to that of the control cycle 14 embryos (A). Bar: 50 μm. **(C-E)** Frames from real-time records showing ventral views of control or cell cycle–arrested embryos with nuclei marked by Histone H2AvD-GFP. Although less robust than controls (C), arrested embryos (D, E) exhibit the hallmark features of gastrulation. Ventral furrow formation is visualized as a central infolding highlighted by rapprochement of nuclei tagged in red and summarized by the paths schematized to the right (green to red). Although only subtle indications of cephalic furrow formation and germband extension are visible in the cycle 12–arrested embryos (E), the paths of marked nuclei show convergent movements of lateral cells associated with ingression of cells in the ventral furrow, and strong or subtle posterior movements reflect germband extension in (D) and (E), respectively. Although embryos arrested in cycle 12 (E) have only rudimentary germband extension, they exhibit distortions in nuclear shapes suggesting physical strain trying to mold a recalcitrant cycle 12 blastoderm. Elapsed time (minutes) from the completion of mitosis 11 is shown at the top-left corner. Green asterisks indicate initiation of the ventral furrow. Bar: 70 μm. GFP, green fluorescent protein.

To determine the extent of MBT in our cell cycle–arrested embryos, we examined postcellularization development. Precisely orchestrated zygotic transcriptional networks subdivide the body plan into segments and guide gastrulation [15, 31, 45]. As part of this program, many transcriptional inputs guide *engrailed* gene expression in segmentally repeated stripes [46, 47]. Antibody staining of control and cell cycle 13 arrested embryos showed remarkably similar patterns of Engrailed protein (Fig 2A and 2B). This observation shows that upon cell cycle arrest, a pre-MBT embryo executes sophisticated transcriptional programing normally exhibited by the post-MBT embryo.

To test for gastrulation, we examined the dramatic reshaping of the embryo during ventral furrow formation and germband extension [48]. Gastrulation in control embryos began with formation of ventral and cephalic furrows approximately 149 minutes after mitosis 11 (Fig 2C), a reference point shared by both the control and arrested embryos. Furrow formation was followed by germband extension (Fig 2C, posterior movement of nuclei highlighted in the schematic). Embryos arrested in interphase 13 initiated gastrulation approximately 133 minutes after mitosis 11 (Fig 2D). These embryos formed relatively normal ventral and cephalic furrows, and their germbands extended robustly. Despite having many fewer nuclei, embryos arrested in interphase 12 still initiated gastrulation (Fig 2E). Although their movements were rudimentary, ventral furrow formation and germband extension could be identified in these embryos. Although poor coordination of movements made the timing of events somewhat ambiguous, features characteristic of gastrulation were seen about 146 minutes after mitosis 11 (Fig 2E). We thus conclude that initiation of gastrulation, an elaborate post-MBT morphogenetic process directed by complex spatially patterned transcription programs, does not require the attainment of a cycle 14 nuclear density or a cycle 14 DNA content.

### Transcriptome dynamics of the embryos arrested in cycle 12

**Fig 3:**
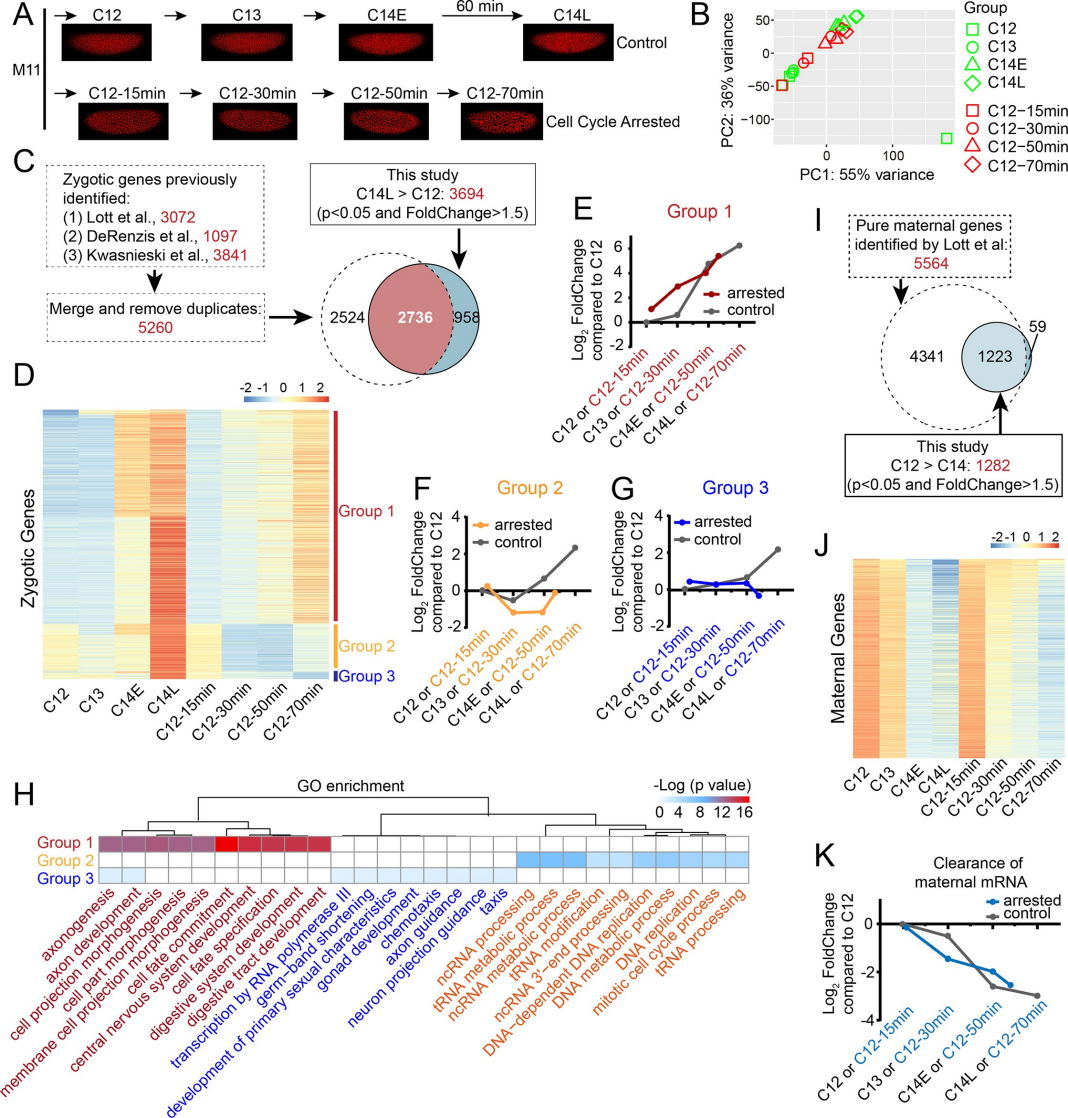
Single-embryo RNA-seq reveals transcriptional dynamics in cell cycle–arrested pre-MBT embryos. **(A)** Images of control and cell cycle–arrested embryos used in RNA-seq experiments. Control and cyclin RNAi-injected embryos are filmed using Histone H2AvD-mRFP and collected at the indicated developmental stages for RNA extraction. C14E is approximately 10 minutes after entering cycle 14, and C14L approximately 70 minutes. Note that the time points in control and experimental groups are not perfectly matched, and the “oldest” cell cycle–arrested embryos (C12: 70 minutes) are still drastically “younger” than the control C14L embryos. **(B)** PCA of RNA-seq data generated from 19 individual embryos. mRNA abundance of 17,735 genes (Log_2_ CPM+1) is used in PCA. Control embryos (green symbols) at different developmental stages form a developmental trajectory from middle left to upper right on the PCA plot, except for one outlier C12 embryo at the lower-right corner, which is removed from subsequent analysis. Embryos arrested in C12 for different amount of time (red symbols) tend to follow the same developmental trajectory of control embryos on the plot. **(C)** Venn diagram showing the previously reported zygotic genes and the ones from this study. The overlapping 2,736 genes are used in subsequent analysis. **(D)** Heatmap showing the relative abundance (scaled Log_2_ CPM+1) of transcripts of the 2,736 genes in control (C12, C13, C14E, and C14L) and cell cycle–arrested embryos (C12: 15, 30, 50, and 70 minutes). RNA-seq data from 2–3 independent embryos at each experimental condition are averaged and used to calculate the Log_2_ CPM+1 for each gene. The 2,736 genes analyzed are categorized into three groups according to their changes in mRNA abundance upon cell cycle arrest. **(E-G)** Line plots of averaged changes of mRNA abundance for genes from different groups. The mRNA abundance for each gene at different experimental conditions is normalized to that of control C12, and the resulting Log_2_ FoldChange is averaged and plotted. Note that the x-axis is meant to represent an approximation of developmental progression; therefore, the C12 70-minute point is not matched to the control C14L point on the plots. This is also true for the plot in (K). **(H)** GO enrichment analysis of the three groups of zygotic genes. The *p*-values are adjusted using the Benjamini-Hochberg method [49]. **(I)** Schematic for identifying the 1,223 maternal genes. **(J)** Heatmap showing the relative mRNA abundance (scaled Log_2_ CPM+1) of the 1,223 maternal genes in control (C12, C13, C14E, and C14L) and cell cycle–arrested embryos (C12: 15, 30, 50, and 70 minutes). The degradation of mRNA for most of the genes still happens upon cell cycle arrest. **(K)** Line plot of averaged changes of transcripts level for the 1,223 maternal genes in control and cell cycle–arrested embryos. Numerical data for panels B, E, F, G, and K can be found in the file S1 Data.xlsx. C12, cycle 12; C14E, control early C14; C14L, control late C14; GO, gene ontology; Log_2_ CPM+1, Log_2_-transformed count per million; MBT, mid-blastula transition; mRFP, monomeric red fluorescent protein; PC, principal component; PCA, principal component analysis; RNA-seq, RNA sequencing; RNAi, RNA interference.

The occurrence of post-MBT developmental events implied the activation of zygotic gene expression, but these studies did not indicate the extent of this activation. To gain a full picture of the dynamics of the transcriptome after cessation of the rapid early cycles, we manually collected embryos that were arrested in cycle 12 (C12) with triple cyclin RNAi for different amounts of time (15, 30, 50, and 70 minutes after completion of mitosis 11), as well as control embryos at different cell cycles, and carried out single-embryo RNA-seq analyses (Fig 3A and S1 Table). We calculated the Log_2_-transformed count per million (Log_2_ CPM+1) for each gene (S2 Table) to allow comparison of their expression levels among different embryos. The normalized mRNA abundance of 17,735 genes from 19 individual embryos was subject to principal component analysis (PCA). A scatterplot of the first and second principal components revealed a simple developmental trajectory (Fig 3B and S1 Data). With the exception of one highly deviant control embryo that was eliminated from further analysis, all control (green symbols) and experimental embryos (red symbols) fell on this linear trajectory, with the only obvious distinction being that the control embryos showed a dramatic and discontinuous transition from C13 to C14, whereas the C12-arrested embryos exhibited a more continuous change with time. This result suggests that a knockdown of the cyclins that arrests the increase in N/C modifies the timing but does not cause a widespread block to the activation of the zygotic gene expression.

To evaluate the activation of zygotic genome, we identified 3,694 zygotic genes that showed up-regulation in control late C14 (C14L) embryos compared to that of C12 (FoldChange > 1.5 and *p* < 0.05) in our RNA-seq data (Fig 3C). Meanwhile, we generated a list of 5,260 zygotic genes that were reported in previous studies [50–52]. We selected the 2,736 genes that were shared by these two lists and used them to probe the ZGA after cell cycle arrest (S3 Table). Whereas in control embryos all the 2,736 genes increased their mRNA abundance as the embryos developed to C14L, in C12-arrested embryos 79.5% of them (2,174/2,736) showed increased mRNA abundance when arrested in C12 for 70 minutes (Fig 3D, group 1). The remaining 20.5% (562/2,736) showed an initial decrease in mRNA abundance upon entry into the C12 arrest (C12: 30 minutes and C12: 50 minutes), reflecting the degradation of maternally contributed transcripts. Interestingly, 487 out of the 562 genes showing this early decrease began to re-accumulate their mRNA at 70 minutes into the C12 arrest (Fig 3D, group 2), suggesting activation of zygotic transcription of these genes and that degradation of maternal mRNA blunted the overall increase in transcript level. Only 75 genes failed to show increasing mRNA levels upon cell cycle arrest (Fig 3D, group 3). We selected representative genes from each group and graphed their expression profiles in control and cell cycle–arrested embryos (S2A–S2C Fig and S1 Data).

To better visualize the overall transcriptional profiles of genes in each group, we normalized their mRNA abundance to that of the control C12 embryos and plotted the mean Log_2_ FoldChange (Fig 3E–3G and S1 Data). The increase of group 1 transcripts started earlier in cell cycle–arrested embryos, suggesting that the experimental cell cycle extension provokes premature activation of transcription (Fig 3E). Despite the delayed activation of transcription in the control embryos, the levels abruptly catch up in cycle 14, perhaps a reflection of their 4-fold higher transcriptional capacity (Fig 3E). This is consistent with what was seen in the PCA plot and the heatmap (Fig 3B and 3D). Group 2 genes exhibited an initial decline in both arrested and control embryos, which we infer represents destruction of maternal transcripts. Subsequently, group 2 transcripts showed an increased abundance (Fig 3F). Genes in group 3, whose transcripts rose in C14L embryos, showed no evidence of an increase in cell cycle–arrested embryos in the time frame examined (Fig 3G).

Next, we performed gene ontology (GO) enrichment analysis on the three groups of zygotic genes (Fig 3H and S3 Table). Interestingly, there were marked distinctions between the groups. Group 1 genes were strongly enriched in GO terms associated with various developmental processes, including cell fate determination and morphogenesis, in agreement with the continued development seen in the C12 arrested embryos (Figs 1 and 2). Group 2 was enriched in genes related to nucleic acid metabolism and cell cycle. Genes in group 3 seemed to function in development as well; however, because only 75 genes were in this group, the GO enrichment was not as accurate.

Since RNA-seq measures both zygotically produced RNA as well as maternally contributed RNA, we also analyzed the degradation of maternal transcripts in the cell cycle–arrested embryos. We selected 1,223 genes that were previously reported as purely maternal [51] and that also showed decreasing mRNA abundance in our RNA-seq data from control embryos (Fig 3I and S4 Table), and we similarly generated a heatmap and an averaged line plot (Fig 3J and 3K, and S1 Data). For the majority of the maternal genes analyzed, the degradation of their transcripts was not affected by the cell cycle arrest. Based on these analyses, we conclude that, to a large extent, both the activation of zygotic genome and the clearance of maternal transcripts can occur without attaining a cycle 14 N/C if the rapid cell cycles are arrested.

Several priming mechanisms have been reported to guide the widespread transcription of the zygotic genome [13]. This includes maternally inherited H4K16ac histone code [53], binding of Zelda and GAGA-factor [54, 55], and preloading of Pol II [27, 28]. We analyzed whether the gene sets used in this study were enriched for any of these features (S2D–S2K Fig and S1 Data). When compared with the maternal gene set, our zygotic gene set was slightly enriched for promoters loaded with Pol II and genes bound by Zelda in the embryo, and reduced for genes bound by GAGA-factor and promoters with H4K16ac modification (S2D–S2G Fig). We wanted to know whether the distinct transcriptional profiles seen in the three zygotic gene groups were due to differences in these priming mechanisms. However, the weak biases detected indicate that the expression behavior of these genes is not fully anticipated by the assessed variables (S2H–S2K Fig).

### Transcripts of “N/C-dependent” genes accumulate below the N/C threshold

**Fig 4:**
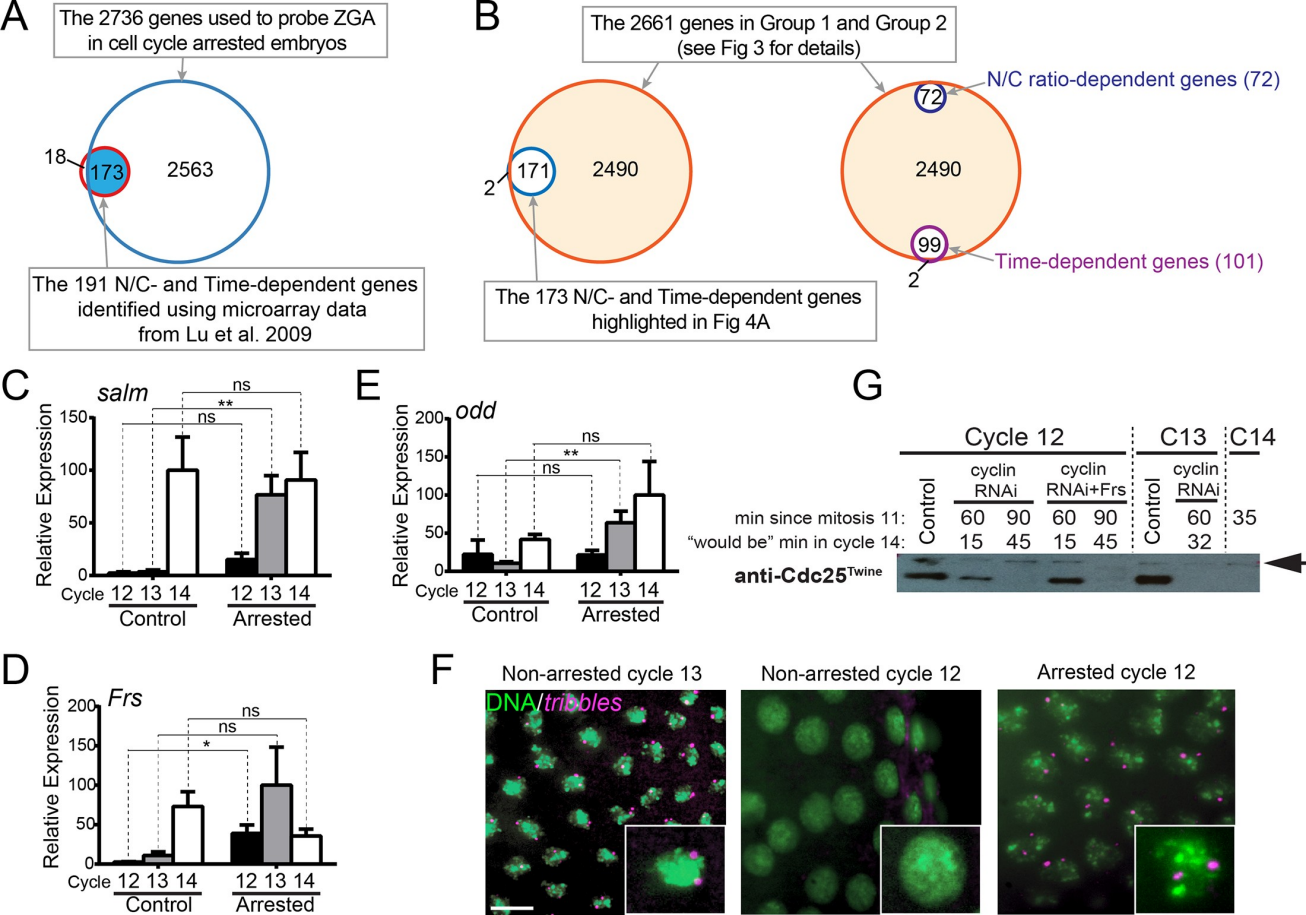
Transcription of “N/C-dependent” genes without reaching the N/C threshold. **(A)** Venn diagrams showing that most of the identified “N/C-dependent” and “time-dependent” genes are present in our gene list used for ZGA analysis. **(B)** Venn diagram showing that all the “N/C-dependent” genes and the majority of the “time-dependent” genes are included in groups 1 and 2. **(C-E)** Single-embryonic qPCR analyses for three “N/C-dependent” genes. Control embryos are in the indicated cell cycle, and experimental embryos are arrested and aged briefly in the indicated cell cycles. More than six independent embryos are used for each condition. The SEM is indicated. Unpaired *t* tests are performed, and *p*-values <0.05 are considered to be statistically significant (*p < 0.05; **p < 0.01). **(F)** Fluorescent in situ hybridization analysis of *tribbles* nascent transcripts. In control embryos, *tribbles* is expressed in cycle 13 (left panel) but not in cycle 12 (middle panel). Upon cell cycle arrest in cycle 12, however, nascent transcripts of *tribbles* become evident (right panel). *tribbles* nascent transcripts are shown in purple, and DNA in green. Bar: 10 μm. **(G)** Single-embryo western blot analysis of Cdc25^Twine^ destruction. Cdc25^twine^ protein is abundant in control cell C12 and C13 embryos (lanes 1 and 6), but absent in C14 embryos (35 minutes into cell C14, last lane). Cdc25^twine^ is also absent in embryos arrested in C13 for 60 minutes (lane 7), or embryos arrested in C12 for 90 minutes (lanes 3 and 5). Cdc25^twine^ persists 60 minutes after arrest in C12 (lanes 2 and 4). Arrow: background band. Numerical data for panels C, D, and E can be found in the file S1 Data.xlsx. Uncropped immunoblot for panel G can be found in S1 Raw Image.tif. C12, cycle 12; Frs, Frühstart; N/C, ratio of nuclei to cytoplasm; ns, not significant; qPCR, quantitative PCR; RNAi, RNA interference; ZGA, zygotic genome activation.

More recent studies of the complexity of the developmental activation of transcription have led to a more nuanced view of N/C regulation of zygotic gene activation in which only specific genes directly respond to N/C [13, 14]. In addition to testing whether widespread genome activation occurs without reaching a cycle 14 nuclear density, we sought to test the proposed role of N/C in the activation of specific genes. By comparing the microarray results of matched diploid and haploid embryos, Lu and colleagues identified 88 “N/C-dependent” and 127 “time-dependent” genes [33]. To analyze these genes in our results, we downloaded the microarray data and used the reported procedure to identify these “N/C-dependent” and “time-dependent” genes. Although our groupings were not identical to those described but not listed in the report by Lu and colleagues, we recovered 81 “N/C-dependent” and 110 “time-dependent” genes (S5 Table). Most of these genes (173/191) fell into the gene list that we used to probe ZGA (Fig 4A). Among these, all “N/C-dependent” genes and all but two of the “time-dependent” genes were transcriptionally activated in the cell cycle–arrested embryos (Fig 4B, S3A Fig, and S1 Data). Thus, genes whose expression was delayed in haploid embryos (the “N/C-dependent” genes) do not require an increase of nuclear DNA content beyond cycle 12 for their transcription when the cell cycle is arrested. This argues that they are not truly N/C dependent. Since haploid embryos do not slow their cell cycles until later, both sets of results can be understood if expression of the “N/C-dependent” category of genes is in fact coupled to cell cycle slowing.

In an effort to detect possible rare genes that are truly N/C dependent but escaped our analysis, we used our RNA-seq data to plot the expression profiles for the 18 genes that were not included in the group that we analyzed for ZGA (Fig 4A). They either showed more ambiguous transcriptional profiles (S3B Fig and S1 Data) or were not detected by our RNA-seq experiment. We also graphed the expression profiles for genes that were individually and stringently verified as “N/C dependent” or “time dependent” in the original study [33]. Whereas the mRNA abundance of a housekeeping gene, *βTub56D*, remained constant, many of the genes in the stringently tested “N/C-dependent” group showed increased transcript levels in our cell cycle–arrested embryos (S3C–S3E Fig and S1 Data). We further selected three stringently defined “N/C-dependent” genes—*spalt major* (*salm*), *Frs*, and *odd skipped* (*odd*)—and used single-embryo quantitative PCR (qPCR) to test if their expression occurred at a sub–cycle 14 N/C when the cell cycle was arrested (Fig 4C–4E and S1 Data). In embryos arrested in cycle 14, the transcripts level of these genes was similar to that in the control cycle 14 embryos. In embryos arrested in cycle 12 or cycle 13, these genes showed some up-regulation, however, to variable degrees.

Our previous work on cell cycle regulation at the time of the MBT uncovered a regulatory circuit in which zygotic expression of *tribbles* contributes to an N/C-sensitive trigger that activates the destruction of Cdc25^Twine^ [37]. As an activating phosphatase that removes the inhibitory phosphorylation from Cdk1, the degradation of Cdc25^Twine^ at the MBT leads to inhibition of cyclin/Cdk1, terminates the rapid nuclear divisions, and allows cellularization (S3G Fig) [36, 37]. This finding suggests that zygotic expression of *tribbles* is a true N/C-dependent transcriptional event and thus stands in contradiction to the observations reported here. We reexamined *tribbles* and *Cdc25* transcription in cell cycle–arrested embryos to further evaluate the coupling to N/C (S3H Fig and S1 Data). There are two *Cdc25* genes, *Cdc25*^*String*^ and *Cdc25*^*Twine*^, in *Drosophila*. *Cdc25*^*String*^ mRNA as well as the protein is degraded early [36, 37], and a similar rapid decrease of *Cdc25*^*String*^ transcript level was observed in embryos arrested in cycle 12. The change of transcripts level for *tribbles* and *Cdc25*^*Twine*^ was less dramatic in cell cycle–arrested embryos; however, zygotic transcription of *tribbles* and mRNA clearance of *Cdc25*^*Twine*^ could still be detected when embryos were arrested in cycle 12 for 70 minutes (S3H Fig). Since some *tribbles* mRNA is loaded into the egg during oogenesis [43, 56], to specifically detect new zygotic expression, we further used an intron probe for in situ hybridization to detect the nascent transcripts (see Materials and methods). Nuclear dots representing nascent transcripts of *tribbles* were absent in control embryos prior to cell cycle 13 (Fig 4F, left two panels). However, when embryos were arrested in cell cycle 12, clear zygotic transcription of *tribbles* was observed (Fig 4F, right panel). Thus, transcriptional activation of *tribbles* does not directly depend on the embryo reaching cell cycle 13.

We next analyzed Cdc25^Twine^ protein destruction in the embryos arrested before the MBT. Cdc25^Twine^ was normally present in cycles 12 and 13 but was no longer detected approximately 35 minutes into cycle 14 (Fig 4G). In our earlier work, we arrested embryos in cycle 12 and aged them to represent times up to 25 minutes into cycle 14 and assessed Cdc25^Twine^ levels. At this time, Cdc25^Twine^ was still detected, and we had concluded that an increase in N/C beyond that of cycle 12 was required for the later destruction of Cdc25^Twine^ [37]. However, on reexamining this, cycle 12–arrested embryos that were aged longer (time equal to 45 minutes into cycle 14) lacked Cdc25^Twine^ (Fig 4G). Thus, we now conclude that embryos do not need to increase N/C beyond a cell cycle 12 level before activating Cdc25^Twine^ degradation, but the destruction is delayed in the arrested embryos. Accordingly, even though Cdc25^Twine^ protein destruction in cycle 14 is delayed to cycle 15 in haploid embryos [37], like other MBT events, it nonetheless occurs in embryos in which N/C increase is blocked in cycle 12 or 13. But we note that embryos arrested with a pre-MBT N/C appear to progress at a somewhat reduced pace, perhaps because of their reduced transcriptional capacity.

### Zygotic transcription is patterned in cell cycle–arrested embryos

**Fig 5:**
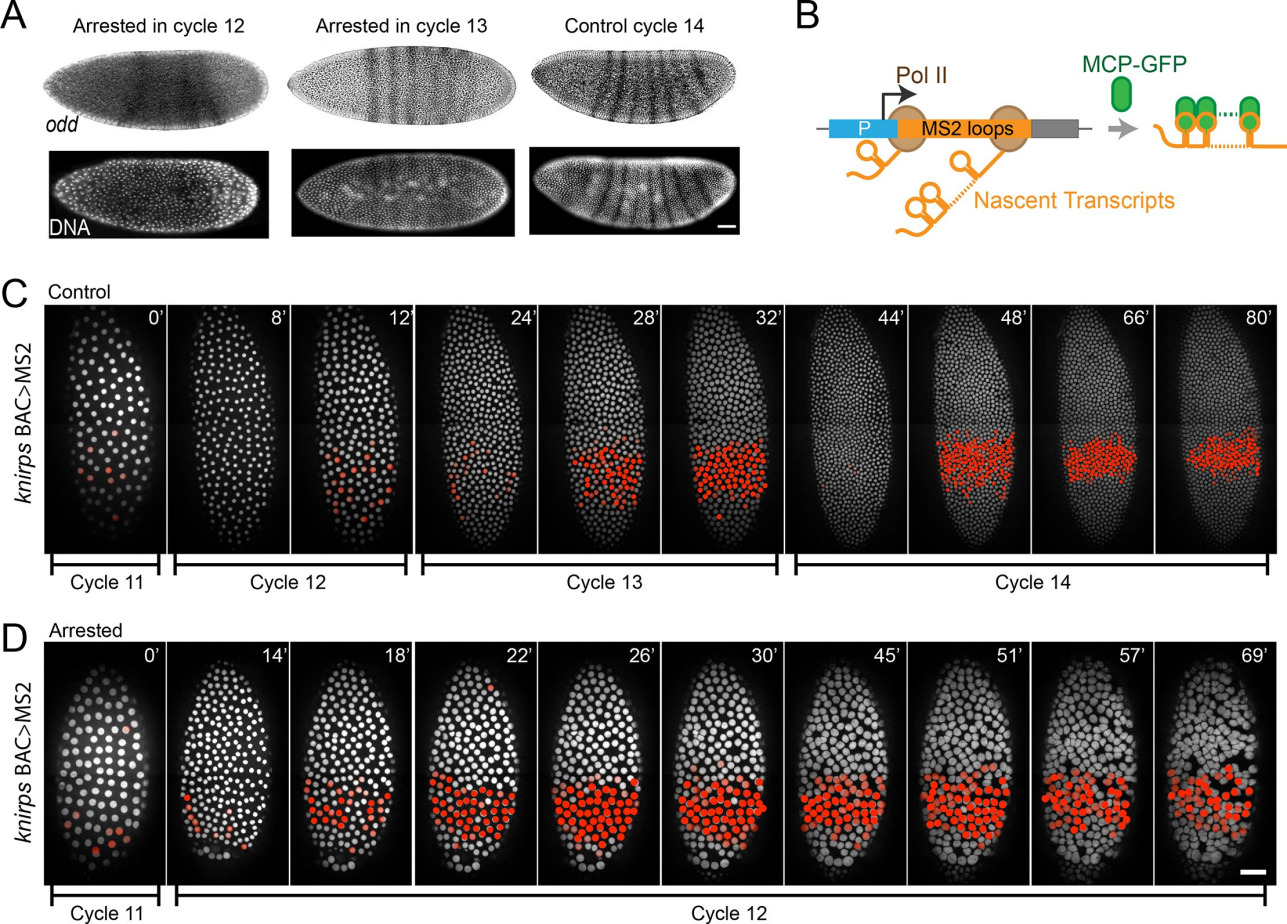
Visualization of the spatiotemporal features of zygotic gene expression by live imaging and in situ hybridization. **(A)** In situ hybridization analysis of *odd*, whose expression is previously thought to be N/C dependent. The striped pattern of *odd* expression is obvious in embryos arrested in cycle 12 or 13 (left and middle panels), although slightly fainter than that in control cycle 14 embryos (right panel). DNA staining is shown below the in situ images to demonstrate the difference in nuclear density (note the DNA signal is quenched by in situ signal). Bar: 34 μm. **(B)** MS2::MCP-GFP system used to visualize nascent transcripts. **(C-D)** Visualization of nascent transcripts of *kni* in control (C) and cell cycle–arrested embryos (D), related to S1 and S2 Movie, respectively. Nuclei with MCP-GFP foci, which represent ongoing zygotic transcription of the reporter gene, are pseudocolored red. Elapsed times (minutes) from the end of interphase 11 are shown at the top-right corner. Nuclei are visualized using mCherry-PCNA. Bar: 50 μm. GFP, green fluorescent protein; *kni*, *knirps*; MCP, MS2 coat protein; N/C, ratio of nuclei to cytoplasm; PCNA, proliferating cell nuclear antigen; Pol II, RNA polymerase II.

Zygotic gene expression is precisely and dynamically controlled both in time and space during early embryonic development [15, 39, 45]. To gain an insight into the patterning of zygotic transcription in cell cycle–arrested embryos, we first used in situ hybridization to detect mature transcripts from a “N/C-dependent” gene *odd*. Expression of *odd* initiates in a single stripe near the anterior end of the embryo early in cell cycle 14 and evolves into a seven-striped pattern over the course of the first hour of cycle 14 [33]. Interestingly, we detected multiple stripes of *odd* expression in embryos arrested in cycle 12 or cycle 13 when these embryos were aged to a stage comparable to control late cycle 14 embryos, although the expression pattern appeared to be less mature in these arrested embryos (Fig 5A). This result suggests that mature transcripts of zygotic genes in cell cycle–arrested embryos mimic the spatial distributions seen in control embryos.

RNA polymerases are densely packed on highly expressed genes, creating concentrated foci of elongating transcripts [39]. When tagged with an RNA sequence recognized by the bacteriophage MS2 coat protein (MCP), these nascent transcripts recruit green fluorescent protein (GFP)-MCP to nuclear foci, representing active transcription at the tagged locus (Fig 5B). This system has been used to visualize the nascent transcripts of *Drosophila* genes involved in pattern formation in the early embryos [57–59]. We selected two genes, *knirps* (*kni*) and *even skipped* (*eve*), which were previously classified as “N/C dependent” because their expression increased dramatically at the MBT and the timing of this change was delayed in haploid embryos (S4A–S4D Fig and S1 Data) [33, 51]. We used their transcriptional reporters to analyze the dynamics and patterning of their zygotic transcription in cell cycle–arrested embryos.

The transcription of *kni*, visualized as distinct MCP-GFP dots inside the nuclei, first became detectable a few minutes after mitosis 10 in control embryos (Fig 5B). In interphase 11 and 12, only rare nuclei showed transient foci late and these foci disappeared abruptly upon entry into mitosis (S1 Movie). About 6–8 minutes into interphase 13 (Fig 5B, 28’), numerous nuclei in the abdominal region of the embryo accumulated intense dots that again vanished at mitosis. In interphase 14, most of the nuclei in this region turned on *kni* transcription 6 minutes after exiting the previous mitosis (Fig 5B, 48’). This transcription was gradually turned off midway through interphase 14 (frames 80–100 in S1 Movie). This shutdown of expression was 72–92 minutes after mitosis 11. In a cycle 12–arrested embryo, *kni* transcription was also weak in interphase 11 during the approach to arrest (Fig 5C). However, about 6–8 minutes after entering the interphase 12 arrest (Fig 5C, 26’), the signal started to increase in the abdominal nuclei, quickly surpassing the signal seen in control cycle 12 embryos, and reached a level comparable to control embryos in late interphase 13 or mid-interphase 14 (Fig 5C). In the supplementary S2 Movie, it can be seen that this localized expression started to decline 72 minutes after mitosis 11 (111’ in the S2 Movie), and the signal was almost gone 24 minutes later.

The transcriptional reporter controlled by the *even skipped* stripe 2 (*eve*2) enhancer also showed dramatically increased transcription in embryos that were arrested in interphase 12 (S4E and S4F Fig). Additionally, the expression-pattern refinement, which occurred during cycle 14 in control embryos, progressed with normal timing to produce two characteristic stripes (S4E and S4F Fig).

In summary, live imaging of nascent transcripts showed that the arrested embryos exhibited high levels of zygotic transcription earlier than control embryos, and the later patterning and declining of transcription also paralleled that of the control embryo. A recent preprint has used the MS2::MCP-GFP system to analyze the zygotic transcription of a handful of genes including *kni* at the MBT as well [38]. By using the integral of fluorescent intensity over time as a surrogate measure of cumulative mRNA production, they similarly found that the zygotic expression level of *kni* is insensitive to N/C but dependent on interphase length. They additionally reported three genes (*gt*, *bnk*, and *Frs*) whose expression was regulated by N/C, and future studies should explore the kinetics of transcriptional activation of these genes in cell cycle–arrested embryos.

## Discussion

We understand little about the control of timing in biology. A century of observing the astonishing speed and temporal precision of early embryogenesis has highlighted the role of timing but yielded little insight. A simple idea that titration of a regulator by an exponentially expanding population of nuclei might serve as a timer has captivated scientists and led to many proposed candidates for the titrated factor [13, 60–66]. Whereas attention has shifted from one candidate titrated factor to another, more continuous progress has been made on a different front: meticulous dissection of the developmental program has given us a detailed context in which to consider the timing of early embryogenesis [4, 31]. The latter gives us some principles that are important in the context of this report. First, events occur quickly. This includes rapid but not coincident onset of transcription of different genes [10, 11, 13, 14]. This is important because the low temporal resolution of many studies have conflated multiple steps into a single proposed transition. Indeed, the complex transcriptional cascades of early development have often been summarized with the low-resolution view of activation of transcription at “the time” of the MBT. Second, studies of cell cycle regulators have demonstrated that cyclin/Cdk1 is down-regulated at the MBT, and multiple genetic perturbations consistently link regulators of this down-regulation to timing of the MBT [4, 5].

**Fig 6:**
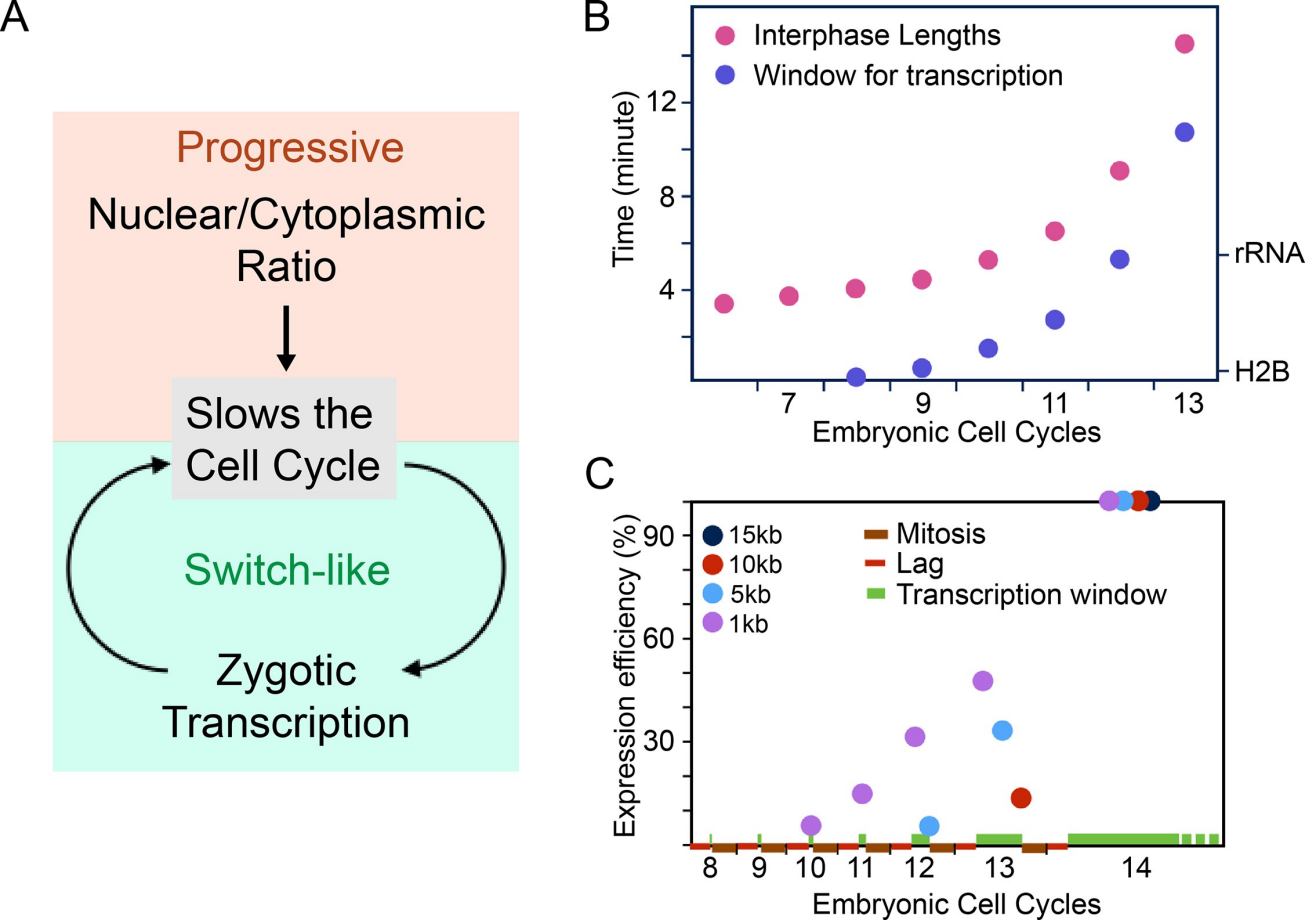
Timing transcriptional activation by relaxing interference from the cell cycle. **(A)** Proposed model in which the N/C input feeds into the MBT through its influence on the cell cycle. During the syncytial cycles, increasing N/C contributes to the increasing duration of the cell cycle. Interphase lengthening allows more time for gene expression and for completion of longer transcripts. Onset of expression of genes such as *tribbles* triggers further cell cycle slowing by engaging a switch-like shutoff of cyclin/Cdk1 by inhibitory phosphorylation that is enhanced by feedback through the expression of additional inhibitors of cell cycle progression. This switch in cyclin/Cdk1 activity, in conjunction with dynamic transcriptional programs, transforms the regulatory architecture of the embryo. **(B)** The duration of interphase of pre-MBT cell cycles (red) with the time available to extend transcripts indicated (blue). The eclipse times for H2B and rRNA are given on the right. As expected, their transcription begins when the window of transcription exceeds the eclipse time. **(C)** Expression efficiency of primary transcripts of four different lengths as function of cell cycle progression. The larger transcription units have a later and more abrupt onset of productive expression. Note that this displays the earliest times that transcripts can be expressed, but their onset can be delayed by additional promoter-specific regulation. Except for cycle 14, which is simply set at 100%, the time-averaged percent expression was obtained by subtracting mitotic time, lag time, and eclipse time from the cell cycle length to determine the time during which complete transcripts can be produced and this is divided by total cell cycle time. This time average estimates a maximal relative efficiency of expression. Numerical data for panels B and C can be found in the file S1 Data.xlsx. MBT, mid-blastula transition; N/C, ratio of nuclei to cytoplasm.

The results here show that when the cell cycle is blocked, many hallmarks of the MBT occur without reaching the normal nuclear density of an MBT embryo. The findings are in accord with more limited studies in which drug-arrested embryos activated transcription [18, 67]. We conclude that events observed in arrested embryos do not require that the N/C increase to a specific MBT threshold. Hence, any coupling of these events to N/C must be due to indirect regulation. As discussed here, this finding leads to a view of the regulation of MBT as a cascade of steps. We suggest that the initial pre-MBT cell cycles slow in an N/C-dependent fashion that allows increasing transcription. This early transcription includes inhibitors of the cell cycle to create a positive feedback loop feeding into a switch-like event that triggers the MBT (Fig 6A).

### N/C input influence pre-MBT cell cycle timing

The pre-MBT cycles are atypical in that they are simple alternations of S phase and mitosis, lacking the two gap phases seen in most cycling somatic cells. The duration of S phase determines the timing of the cell cycle at this stage. Lengths of S phase extend progressively prior to the MBT, from as short as 4 minutes at pre-blastoderm stage to 7 minutes at cycle 10 and 15 minutes at cycle 13. The gradually extending S phases provide a broader transcriptional window as the number of nuclei increases in each successive cycle (Fig 6B and S1 Data).

The increase in S phase duration is due to a reduction in the synchrony with which different regions of the genome replicate [26]. In the early cycles, high levels of interphase cyclin/Cdk1 promote early firing of all origins [35], and all regions of the genome are replicated at the same time [68]. As the cycles progress, the replication of the highly repetitive satellite sequences (30% of the genome) is incrementally delayed [26], in response to a progressive decline in cyclin/Cdk1 activity [23, 35, 69, 70].

A seminal analysis of the key regulators of cyclin/Cdk1 in staged single embryos revealed three pathways that contribute to progressive down-regulation of cyclin/Cdk1 in the pre-MBT blastoderm cycles [23]. First, in the earliest mitotic cycles, destruction of mitotic cyclins is so limited that there is no observed decline. By cycle 10, a modest decline in cyclin levels occurs at anaphase. This decline increases progressively in subsequent mitoses. By mitosis 13, 80% of the mitotic cyclin is degraded. Second, following exit from each of the blastoderm mitoses, Cdk1 transiently loses its activating phosphate on T-161; this loss is more complete and restoration of the activating phosphate is increasingly delayed in successive cycles. Third, at each successive cycle a checkpoint pathway operating through a kinase cascade inhibits cyclin/Cdk1 to delay mitosis and incrementally extend the early cycles [24, 71]. Recently, this gradual down-regulation of cyclin/Cdk1 activity in pre-MBT embryos has been elegantly recapitulated by live-imaging studies with biosensors [69, 70]. These high-spatial resolution studies also uncovered a crucial role of protein phosphatase 1 (PP1) in coupling the dynamics between nuclei and the cortex, revealing an urgent need for better understandings of protein phosphatases, such as PP1 and PP2A, in regulating cyclin/Cdk1 activity (e.g., Cdk1’s activating phosphorylation on T-161) during embryonic development [5, 72–74].

Despite incomplete understanding of the molecular mechanisms, the process of cyclin/Cdk1 down-regulation is coupled to the rising N/C [4, 17]. Cyclins’ destruction at mitotic exit is restricted to the mitotic spindle during pre-MBT cycles [70, 75]. As the number of mitotic spindles increases with each cycle, cyclin destruction progressively increases. Once the decline in cyclins becomes apparent, Cdk1 starts to lose its activating phosphorylation on T-161 perhaps via PP1 or PP2A. Hence, the increasing N/C could impact both cyclins levels and activating phosphorylation of Cdk1. On the other hand, work in both *Xenopus* and *Drosophila* showed that Chk1, a checkpoint kinase acting in a pathway that suppresses cyclin/Cdk1, is progressively activated in early cycles in response to increasing DNA [66, 69, 76]. In line with this, Chk1 kinase, encoded by *grapes*, together with the other genes in this checkpoint pathway, is responsible for a progressively increasing delay in entry into mitosis in *Drosophila* embryos [24, 69, 71, 77, 78]. Another report further suggested that activation of the checkpoint in *Drosophila* is coupled to N/C and that the activating signal is substantially dependent on complexes formed on promoters early in cycle 13 [28], whereas other work suggested that depletion of maternally supplied deoxynucleotide triphosphates (dNTPs) and titration of free histones contribute to progressive activation of the checkpoint [64–66, 79]. Although many unanswered questions remain, it appears that N/C has multiple inputs each progressively strengthening a different regulatory circuit that diminishes interphase cyclin/Cdk1 activity and gradually extends the pre-MBT cell cycles.

### A simple model for “activation” of transcription by cell cycle slowing

Mitosis is a disruptive event that interferes with many cell biological processes, including transcription [1]. Transcription is not only inhibited during mitosis, it is aborted [39]. Incomplete nascent transcripts are abandoned and degraded. As a result, when transcription begins in the next interphase, completed transcripts cannot be produced until RNA polymerase initiates and then traverses the entire transcription unit (TU). Mitosis constitutes a deadline for transcription: all transcripts that are not completed prior to the next mitosis will be aborted [39, 80, 81]. We refer to a time delay between mitosis and initiation of transcription as a lag, and the time between initiation of transcription and production of complete transcripts as the eclipse time (an analogy to the eclipse period between viral infection and production of mature viruses). The eclipse time is proportional to the length of the primary TU (kb × 0.7 minutes/kb). Mitosis takes about 5 minutes, and real-time detection of nascent transcripts shows a lag of about 4 minutes (Fig 5) [57]. Subtracting the duration of mitosis and the lag from cell cycle duration gives a transcriptional window, the period of opportunity to extend transcripts (Fig 6B). The transcriptional window for cycle 8 (approximately 12 s) is compatible with completion of only extremely short RNAs (approximately <200 bp). The window increases, at first only incrementally, then more dramatically as the cycle is prolonged (Fig 6C, green bars on the x-axis). Each successive cycle is compatible with expression of longer primary transcripts.

Even when transcripts can first be completed, their expression will be inefficient because production of complete transcripts only occurs toward the end of the transcriptional window. Subtracting the eclipse time for TUs of different length from the transcriptional window, we estimate the duration of time during which completed transcripts can be made. Comparing time of production of complete transcripts to the duration of each cell cycle gives the percent of the time that TUs of different sizes can be expressed (Fig 6B and 6C, and S1 Data). This model for mitotic inhibition of transcription gives a profile for developmental “activation” of transcription that parallels observed increases and the shift in the size of expressed genes [18, 20]. Accordingly, we suggest a fundamental antipathy between cell cycling and transcription that is especially dramatic in the early cycles.

The proposed linkage of transcript accumulation with cell cycle prolongation suggests that the connection between transcriptional activation and N/C might be secondary to N/C-dependent prolongation of the cell cycle. This is in line with our finding that premature slowing of the cell cycle uncouples transcriptional activation from the N/C. Furthermore, the proposed coupling of cell cycle duration and transcriptional competence also explains the delayed onset of zygotic transcript accumulation in haploid embryos. The duration of cycles 13 and 14 in haploid embryos was much reduced and was similar to cell cycles 12 and 13 of diploid embryos [18]. Thus, at a given cell cycle, the haploid embryos will have shorter window available for transcript extension, and the timing change is shifted by one cell cycle at just the time that normal embryos exhibit a rise in transcription. Finally, our demonstration here that arrest of the cell cycle gives rise to widespread release of constraints on transcription suggests that a general inhibition of transcription during the pre-MBT blastoderm cycles is due to the disruptive influence of frequent mitoses [18, 39]. Consequently, we suggest that N/C increase triggers the early slowing of the cell cycle and thereby indirectly guides the initial onset of transcription.

In addition to the lengthening of the transcription window, there are many changes accompanying the approach to MBT that might influence the activation of the zygotic genome. A large increase in the recruitment of Pol II to promoters in cell cycle 14 might reflect a fundamental change in the transcription machinery [27, 28]. Alternatively, these large accumulations of polymerase might be slow processes that can only occur upon lengthening of the cell cycle. Perhaps the latter explanation is favored by our finding that cell cycle arrest by cyclin knockdown is sufficient to activate post-MBT expression, but it is also possible that knockdown of cyclin/Cdk1 activity triggers modification of the transcriptional machinery. It has been noted that promoters expressed before the MBT have a dearth of paused polymerase, a feature that is expected to enhance the speed of activation, which would impart these early genes with short lag in their postmitotic activation [27, 82, 83].

We propose that evolution has adapted to the constraints of the fundamental antipathy between the cell cycle and transcription by giving genes properties allowing expression at different stages of the developmental program. In this view, rapid onset of transcription and small size give early expressed genes the capacity to be expressed in the very early stages of the progressive prolongation of early cycles. These early expressed genes might then reinforce cell cycle slowing (e.g., *tribbles* and *Frs*) and thus promote the abrupt extension of cell cycle 14 (Fig 6A), when genes lacking the specializations of the early genes begin to be expressed.

## Materials and methods

### Fly stocks used

The fly stocks used were *Sevelen* (WT), H2AvD-GFP, H2AvD-mRFP, H2AvD-mRFP;Da-gal4, UASp-YFP-Anillin/MKRS, GFP-Fzr/Cyo;H2AvD-mRFP/TM6B, *grp*^*06034*^, H2AvD-GFP/Cyo-DTS513, *yw*;Histone-RFP;MCP-NoNLS-GFP, *eve2*-MS2-yellow, *kni* BAC>MS2.

### Embryo injections

H2AvD-GFP or H2AvD-RFP embryos were injected lengthwise three times along the dorsal side on a dissecting scope using air pressure. The embryos were then imaged with either a spinning disc confocal microscope (Perkin Elmer) or a laser scanning microscope (LSM880, Zeiss). Cell cycle stage was confirmed by measuring internuclear distance. Double injection of cyclin RNAi and Frs required first the injection of dsRNA targeting all three mitotic cyclins followed by visualization on microscope. Upon entry into the desired cell cycle for cell cycle arrest, the embryos were removed and injected with Frs, using the initial injection points. Embryos were then returned to the microscope and the recording resumed.

### dUTP-546 injections

Staged embryos were injected with cyclin RNAi on a schedule to arrest them in cycle 12. In a second injection, approximately 10 minutes after entering cell cycle 12, they were co-injected with dUTP-546 (Alexa) and Frs. Note that these embryos would be completing S phase 12 by the time of this second injection. Robust incorporation would indicate re-replication in arrested embryos. These embryos were then allowed to age for 2.5 hours before being removed from glue, devitellinized by hand, washed five times for 20 minutes in PTX (0.1% Triton-X in 1X PBS), and fixed. Embryos arrested in cycle 13 were injected with dUTP-546 approximately 15 minutes after entry into cell cycle 13 and allowed to age for 1 hour before removal and fixing, as described above. In a control labeling of the last S phase, embryos were treated as above but injected with dUTP-546 one cycle prior to arrest. These embryos were then aged for 1 hour before removal, washing, and fixing, as described above. All embryos were then stained with WGA-488 (Life Technologies) and washed with PTX before being visualized on a spinning disc confocal using Volocity software (Perkin Elmer).

### Single-embryo RNA extraction, library preparation, and sequencing

H2AvD-RFP flies were used in the RNA-seq experiment. Control embryos or embryos that were arrested with triple cyclin RNAi without Frs were aged and collected for RNA extraction. The RNA extraction from single embryos was done with TRIzol (Life Technologies #87804) according to the manufacturer’s protocol and a previous report [51]. We obtained 80–150 ng of total RNA from each embryo. Total RNA was made into libraries for sequencing using the mRNA-Seq Sample Preparation Kit (Illumina) and sequenced on an Illumina Hiseq platform (Novagene). Sequencing data are available at the GEO database (accession number GSE154502).

### Reads clean, mapping, qualification, and analysis

We first cleaned the raw reads using trim_galore (version TrimGalore-0.6.0). Then, reads from each RNA-seq sample were mapped to the reference *Drosophila melanogaster* genome (dm6; version BDGP6) downloaded from Ensembl [84] using STAR (version STAR-2.5.3a) [85]; key alignment scripts were as follows: “—outFilterMismatchNoverLmax 0.04—outSAMtype BAM SortedByCoordinate—outFilterMultimapNmax 1—outMultimapperOrder Random—outSAMmultNmax 1.” Finally, transcripts abundance for annotated RNAs were called by featureCounts of Subread package (version subread-1.6.0-Linux-x86_64) [86], and only uniquely aligned reads were included. Statistics on the sequencing and mapping were reported in S1 Table.

To quantify the transcripts abundance, we used the function “fpm” in R package DEseq2 to calculate counts per million mapped fragments (CPM) for each gene in all the samples, and then we transformed the CPM to Log_2_ scale (CPM+1 was used to avoid Log_2_ 0) (S2 Table). To perform PCA analysis, we used the function “plotPCA” in R package DEseq2. The scatterplot was generated with the R package ggplot2 [87]. To generate the heatmaps, we first used the function “scale” in R for each gene and then used the package pheatmap. The GO enrichment analysis was performed by the R package clusterProfiler [88]. Venn diagrams were prepared with the R package Vennerable and venn.

### Single-embryo qPCR analysis

Embryos were individually visualized and staged and cDNA was made from mRNA extracted from single embryos or pools of multiple embryos. Differences between the protocol for single and multiple embryos are noted. Each embryo was removed from glue into heptane using a tungsten needle, and removal of correct embryo was confirmed using a dissecting scope. Each single embryo was then transferred to 150 μL of TRIzol Reagent (Life Technologies #87804) and flash-frozen in liquid nitrogen. Using an RNAse-free pestle (Kimble Chase #061448), the TRIzol/embryo mixture was ground up until the TRIzol was melted. For collecting mRNA from multiple embryos, embryos were smashed in TRIzol using the pestle without freezing. TRIzol (250 μL) was added and allowed to incubate at room temperature for at least 5 minutes. Chloroform (80 μL) was then added and the tube was shaken well. Extracts were incubated at room temperature for 3 minutes and centrifuged at 12,000*g* for 15 minutes at 4°C. The clear, aqueous phase was transferred to a new tube. Chloroform (80 μL) was added and the tube was shaken well. The tube was then allowed to incubate for 3 minutes at room temperature. The samples were again spun at 12,000*g* for 10 minutes at 4°C. The top, aqueous phase was transferred to a new tube and 1 μL of 10 mg/μL RNAse-free glycogen was added and mixed. Isopropanol (200 μL) was then added and incubated for 10 minutes at room temperature. The samples were then spun at 12,000*g* for 10 minutes at 4°C. The pellet was washed with 200 μL of 70% ethanol and then spun at 7,500*g* for 5 minutes at 4°C. The pellet was allowed to air-dry before suspending it in 20 μL DEPC-treated water. The RNA was then treated with TURBO DNA-free kit (Life Technologies) following the user guide protocols. A Sensifast cDNA Synthesis Kit (Bioline #65053) was used to make cDNA. The RNA isolate (15 μL) was added to 4 μL of 5X Buffer and 1 μL RT polymerase. The product’s protocols were followed to produce the cDNA in a thermocycler.

This cDNA was then used as a template for triplicate reactions of *odd*, *salm*, *Frs*, and *βtub56D* using a Sensifast SYBR Lo-Rox kit (Bioline). A Stratagene MX3000 was used to perform the qPCR. Expression levels for each gene was normalized to *βtub56D* and relative expression levels of each gene was then calculated in Microsoft Excel.

*Odd1* F: GATACAAGTGCTAAGCCAAAGT

*Odd1* R: CCTTGATCACTATGAAATCCTC

*Salm3* F: AAATATGGCATTGTCAAACAG

*Salm3* R: GGTATGCTCTGCTCTGAAGT

*Frs3* F: AGCAAATCAGCAACGTCAAGC

*Frs3* R: GGAATACTTCTTGCTGTCCAGG

*βtub56D1* F: GTTGTTGTTCGACTGCTATAAG

*βtub56D1* R: GACGCCTCATTGTAGTACAC

### DIG-labeled probe production

Primers were designed to recognize approximately 200-bp regions of *odd skipped* transcript sequence (using www.Flybase.org; see primer sequences below). A first round of standard PCR using Velocity Taq Polymerase (Bioline) was performed using embryonic cDNA collected from a large number of embryos aged to 2–3 hours after egg deposition, as described above. Products were checked for correct size by agarose gel electrophoresis. These PCR products were used as template for the next round of PCR. The PCR DIG Probe Synthesis Kit (Roche) was used to make DIG-labeled probes. For a 20 μL reaction, each reaction included 11.98 μL water, 0.6 μL DMSO, 4 μL buffer, 1 μL dNTP mix, 1 μL DIG dNTP mix, 0.5 μL template cDNA, 0.8 μL reverse (R) primer, and 0.12 μL Velocity Taq Polymerase. These second-round products were precipitated using standard methods and quantified using a NanoDrop.

*Odd1* F: GATACAAGTGCTAAGCCAAAGT

*Odd1* R: CCTTGATCACTATGAAATCCTC

*Odd2* F: GATGAAAAATCCAATGAAAAGT

*Odd2* R: TGGGCTACTACGACTATAAGGT

*Odd3* F: AAAGTCAAATAGCAGAGGAAAA

*Odd3* R: TGGGCTACTACGACTATAAGGT

*Odd4* F: ATGAGCAGATAGATTGAAGGAC

*Odd4* R: ATTGGGATCTGCTACATAGAAC

*Tribbles1* F: TATGCCTGACTTATACCAACTC

*Tribbles1* R: CAATGTTACCCACAACGACG

*Tribbles2* F: TGCAATCGAATCACCAATAGG

*Tribbles2* R: AGCTACAAAGCTAATACACC

*Tribbles3* F: AATTGCCATTCAGTGAGCAAG

*Tribbles3* R: TGTGGCAATTTCTAGCTTCC

### *tribbles* in situ hybridization

Embryos were staged, injected, collected, and fixed as described above for specific cell cycle arrests. DIG-labeled probes were designed as described above but directed to the intron of the *tribbles* gene. Embryos were then prepared for in situ hybridization as described above, except a sheep anti-DIG-HRP antibody (Roche) was used. A Tyramide Amplification Kit (Life Technologies #T-20913) was used with provided instructions to develop the signal. Embryos were stained with Hoechst (1:1,000 in PTX) for 1 minute, washed with PTX five times for 2 minutes, and transferred to a microscope slide to be visualized.

### Twine protein western blots

Embryos were staged, injected, and collected as described above. Embryos of interest were removed from the coverslip using a tungsten needle and transferred to a 1:1 mixture of heptane and methanol in a PCR tube. The tube was shaken and kept on ice until ready to proceed. Each embryo was then transferred to a separate and new PCR tube with glass beads and 20 μL of 2X SDS sample buffer was added. Each tube was vortexed for 8 seconds and then boiled for 8 minutes. After boiling, the sample was transferred to an Eppendorf tube by using a heated needle to poke a small hole in the bottom of the PCR tube and centrifuging the PCR tube in an Eppendorf tube for a short amount of time. Ten microliters of each sample was then loaded into a Mini-PROTEAN TGX precast gel (BioRad) and ran for 1 hour at 200 V. The gel was transferred to a membrane for 1.5 hours at 80 V in the cold room. The blot was then blocked for 1 hour using 3% milk in PBST (0.1% Tween20 in 1X PBS) and incubated with rabbit anti-Cdc25^Twine^ (1:1,000) overnight. The primary mixture was poured off and then incubated with donkey anti-rabbit HRP (Jackson Labs; 1:10,000) for 1 hour at room temperature. The blot was then visualized using SuperSignal West Femto Maximum Sensitivity Substrate (ThermoScientific #34095) and Phenix Premium X-Ray Film.

### Visualizing zygotic transcription

Transcriptional reporter lines of *knirps* (*kni*) and *eve2* were used as previously described [57–59]. Briefly, female virgins expressing MCP-GFP in the germlines were crossed with males containing the transcriptional reporters. The yielded embryos were then dechorinated, injected with cyclin RNAi, and used in live-imaging experiments as previously described [35].

### In situ hybridization

Arrested samples for in situ hybridization were collected two different ways. With the first, larger batches of embryos were collected in short windows of time (10–15 minutes), aged, aligned and transferred to a microscope slide, injected, and then aged again for 1–1.5 hours. These embryos were then washed off the glue on the microscope slide using heptane. The embryos were then fixed in a combination of 5% formaldehyde solution and heptane in 1.5-mL microfuge tubes for 15 minutes. After fixation, embryos were washed with 1X PBS and devitellinized by hand using a tungsten needle. The embryos were transferred and stored in methanol until ready for use in in situ hybridization. For the second method, embryos were collected and aged as before but transferred to a coverslip and taped to a custom-made metal coverslip holder. After injection, embryos were monitored on a spinning disc confocal microscope using Volocity software. After allowing these embryos to age for an appropriate amount of time, embryos of known cell cycle arrest and aging duration were removed from the glue using a tungsten needle and transferred to heptane. Removal of the correct embryo was confirmed by dissecting microscope. The embryos were then fixed and devitellinized by hand.

Embryos ready for in situ hybridization were gradually rehydrated through a methanol series into PTX. Embryos were then postfixed in 7% formaldehyde for 20 minutes and then washed for 5 minutes, four times in PTX. A 1:1 mixture of hybridization buffer (5 mL Deionized Formamide, 2.5 mL 20X SSC, 100 μL 10 mg/mL tRNA, 40 μL 10 mg/mL heparin, 10 μL Tween20, and 2.34 mL water) and PTX was added to embryos for 10 minutes at 48°C. After that, hybridization buffer was added to the embryos for 10 minutes at 48°C. Fresh hybridization buffer was then added and the embryos were incubated at 48°C for 1 hour. DIG-labeled probes (5 ng/μL) were incubated at 95°C for 10 minutes and then place on ice. Once the embryos had incubated for 1 hour at 48°C, the hybridization buffer was removed and the probes were added. The embryos were then incubated overnight at 48°C. After the overnight incubation, the probes were removed for reuse. The embryos were washed with prewarmed hybridization for 20 minutes, then with a prewarmed 1:1 mixture of PTX and hybridization buffer for 20 minutes, then prewarmed PTX three times for 20 minutes. The embryos were then blocked with 10% Normal Donkey Serum (NDS; Jackson Labs) in PTX for 1 hour at room temperature. Sheep anti-DIG-alkaline phosphatase fab fragments (Roche; 1:500) were then added to the embryos in 10% NDS in PTX for 1 hour at room temperature, rocking. Embryos were rinsed five times for 10 minutes each with PTX. Embryos were then rinsed with Rinse Solution (1 mL 1 M NaCl, 500 μL 1 M MgCl2, 1 mL Tris [pH 9.5], 10 μL Tween20, 7.5 mL water) twice for 2 minutes. NBT/BCIP Stock Solution (Roche) in Rinse Solution (1:100) was then added to the embryos for 10–30 minutes. The embryos were rinsed with PTX five times 5 minutes each, stained with Hoechst (Molecular Probes; 1:1,000) in PTX, rinsed again with PTX five times 1 minute each, and then transferred to a microscope slide to be visualized.

### Visualizing gastrulation

H2AvD-GFP embryos were collected, injected, and monitored via microscopy as described above. To image ventral furrow formation, embryos were aligned ventral side down and injected lengthwise. Injected and control embryos were imaged once per minute for the duration of each movie made. Times for different MBT events were aligned to completion of mitosis 11.

### Engrailed visualization

Embryos were staged, injected, collected, and fixed as described above. Once the embryos were devitellinized by hand, they were transferred to PTX. The embryos were blocked in 1% BSA for 1 hour. Embryos were then incubated with rabbit anti-Engrailed (1:1,000) for 2 hours at room temperature. Embryos were rinsed five times with PTX, 10 minutes each. Embryos were then incubated with goat anti-rabbit conjugated to Alexa546 (1:1,000) for 1 hour at room temperature. Embryos were rinsed five times with PTX, 10 minutes each. Embryos were stained with Hoechst (Molecular Probes; 1:1,000 in PTX) for 1 minute and rinsed with PTX five times for 2 minutes. Embryos were then transferred to a microscope slide to be visualized.

## Supporting information

S1 Fig Combination of cyclin RNAi with Cdk1 inhibitor Frs arrests both nuclear and centrosome cycles. **(A)** No DNA re-replication after cell cycle arrests. Almost no deoxynucleotide incorporation (purple) is observed when dUTP-546 is injected approximately 10 minutes (cycle 12) or 15 minutes (cycle 13) after entry into an arrested cell cycle. Bar: 10 μm. **(B)** Arresting the centrosome cycle by cyclin RNAi and Frs protein. When nuclear cycles are arrested by cyclin RNAi, centrosome cycles continue, resulting in about eight centrosomes per nucleus (middle panel, marked by arrows). When embryos are injected with cyclin RNAi and Frs protein, both centrosome and nuclear cycles are stopped, and the centrosome to nucleus ratio remains as 2 (top and bottom panels, marked by arrows). Numbers at the top-right corner in each image are elapsed time (minutes) from the completion of mitosis 10 (top and middle panels) or mitosis 11 (bottom panel). Bar: 5 μm. Frs, Frühstart; RNAi, RNA interference.
(TIF)

S2 Fig Temporal profiles of total mRNA for representative genes from different groups and the enrichment analyses for factors regulating ZGA. **(A-C)** Line plots of the Log_2_ CPM+1 for representative genes from group 1 (A), group 2 (B), and group 3 (C). Related to Fig 3D. **(D-G)** Differences in RNA Pol II loading, binding of Zelda and GAGA factors, and H4K16ac histone modification between the maternal and zygotic genes analyzed in Fig 3. The zygotic gene list contains more genes that are positive for RNA Pol II loading and Zelda binding (Fisher’s test, *p* < 0.0001), whereas the maternal gene list includes more genes that have GAGA binding and H4K16ac modification (Fisher’s test, *p* < 0.05 and *p* < 0.0001, respectively). **(H-K)** Comparison of RNA Pol II loading, Zelda and GAGA bindings, and H4K16ac modification states among the three groups of zygotic genes. Differences in Pol II loading, GAGA binding, and H4K16ac modification are observed between group 1 and group 2 (Fisher’s test, *p* < 0.001, *p* < 0.0001, and *p* < 0.0001 respectively). Numerical data for panels A-K can be found in the file S1 Data.xlsx. Log_2_ CPM+1, Log_2_-transformed count per million; Pol II, RNA polymerase II; ZGA, zygotic genome activation.
(TIF)

S3 Fig Temporal expression profiles for the indicated genes. **(A)** The two “time-dependent” genes that are not increasing their transcripts levels upon cell cycle arrest. **(B)** Transcriptional profiles for the “N/C-dependent” and “time-dependent” genes that are not included in our analysis (see Fig 4A). **(C)** The abundance of transcripts of *βTub56D* are comparable among embryos from different experimental conditions. **(D-E)** The previously verified “N/C-dependent” genes (*gt*, *Frs*, *salm*, *sog*, *odd*, and *opa*) and “time-dependent” genes (*ilp4*, *CG5888*, *spo*, and *sna*) increase their mRNA abundance upon cell cycle arrest. **(F)** *kni* and *eve*, the two genes selected for the subsequent nascent transcript imaging experiments, show increased mRNA abundance when the cell cycle is arrested. **(G)** Developmental control of cyclin/Cdk1. The inhibitory phosphorylation of Cdk1 is added by Wee and Myt1 kinases and removed by Cdc25. There are two *Drosophila* Cdc25, Cdc25^String^, and Cdc25^Twine^. The zygotic transcription of *trbl* triggers the destruction of Cdc25^Twine^ at the MBT, which results in inactivation of cyclin/Cdk1 and hence cell cycle slowing. **(H)** The transcriptional profiles of key regulators of the cell cycle in development, *trbl*, *stg*, and *twe* in control and cell cycle–arrested embryos. Numerical data for panels A-F and H can be found in the file S1 Data.xlsx. *eve*, *even skipped*; *Frs*, *Frühstart*; *kni*, *knirps*; MBT, mid-blastula transition; N/C, ratio of nuclei to cytoplasm; *odd*, *odd skipped*; *salm*, *spalt major*; *stg*, *Cdc25*^*String*^; *trbl*, *tribbles*; *twe*, *Cdc25*^*Twine*^.
(TIF)

S4 Fig Refinement of gene expression pattern in control and cell cycle–arrested embryos. **(A)** Increase of *kni* transcript level in cycle 14. The data are adopted from Lott and colleagues [51]. x-Axis is embryonic cell cycles (note that cycle 14 is roughly divided into four stages). **(B)** The “N/C dependency” of the expression of *kni*. The expression of *kni* gene is delayed by one cell cycle in the haploid embryos. The data are adopted from Lu and colleagues [33]. **(C)** *eve* mRNA level during early embryonic development. **(D)** The expression of *eve* in diploid and haploid embryos. **(E)** Visualization of *eve2* transcriptional activity in control embryos. **(F)** *eve2* transcriptional activity in embryos arrested in cycle 12. Note that a similar but less precise expression pattern is established in the arrested embryos (82’ in panel F versus 80’ in panel E). Nuclei are visualized using mCherry-PCNA. Bar: 50 μm. Numerical data for panels A, B, C, and D can be found in the file S1 Data.xlsx. D13, diploid cycle 13 embryos; *eve2*, *even skipped* stripe 2; *Frs*, *Frühstart*; H14, haploid cycle 14 embryos; *kni*, *knirps*; N/C, ratio of nuclei to cytoplasm; PCNA, proliferating cell nuclear antigen.
(TIF)

S1 Table Statistics of the RNA-seq and reads mapping. RNA-seq, RNA sequencing.
(XLSX)

S2 Table Log_2_ CPM+1 for all the 17,735 genes detected in our RNA-seq experiments. Log_2_ CPM+1, Log_2_-transformed count per million; RNA-seq, RNA sequencing.
(XLSX)

S3 Table Log_2_ CPM+1 for the 2,736 zygotic genes used in ZGA analyses after cell cycle arrest, and the results for GO enrichment analysis on the three groups of zygotic genes. GO, gene ontology; Log_2_ CPM+1, Log_2_-transformed count per million; ZGA, zygotic genome activation.
(XLSX)

S4 Table Log_2_ CPM+1 for the 1,223 maternal genes used in the analysis of maternal mRNA clearance after cell cycle arrest. Log_2_ CPM+1, Log_2_-transformed count per million.
(XLSX)

S5 Table The identified N/C- and time-dependent genes. N/C, ratio of nuclei to cytoplasm.
(XLSX)

S1 Movie MS2::MCP-GFP transcriptional reporter for *kni* in control embryos. GFP, green fluorescent protein; *kni*, *knirps*; MCP, MS2 coat protein.
(MOV)

S2 Movie MS2::MCP-GFP transcriptional reporter for *kni* in cell cycle–arrested embryos. GFP, green fluorescent protein; *kni*, *knirps*; MCP, MS2 coat protein.
(MOV)

S1 Data Excel file containing numerical values that underlie the summary data displayed in Fig 3B, 3E, 3F, 3G and 3K; Fig 4C, 4D and 4E; Fig 6B and 6C; S2A–S2K Fig; S3A–S3F and S3H Fig; S4A–S4D Fig. (XLSX)

S1 Raw Image Uncropped immunoblot image for Fig 4G. (TIF)

## Abbreviations

C14E: control early cycle 14
C14L: control late cycle 14
dNTP: deoxynucleotide triphosphate
dsRNA: double-stranded RNA
*eve*2: *even skipped* stripe 2
Frs: Frühstart
GFP: green fluorescent protein
GO: gene ontology
*kni*: *knirps*
*eve*: *even skipped*
Log_2_ CPM+1: Log_2_-transformed count per million
MBT: mid-blastula transition
MCP: MS2 coat protein
N/C: ratio of nuclei to cytoplasm
ns: not significant
*odd*: *odd skipped*
PCA: principal component analysis
Pol II: RNA polymerase II
PP1: protein phosphatase 1
qPCR: quantitative PCR
RNAi: RNA interference
RNA-seq: RNA sequencing
*salm*: *spalt major*
TU: transcription unit
ZGA: zygotic genome activation
