# Supplementary figures and images for: Interphase-arrested Drosophila embryos activate zygotic gene expression and initiate mid-blastula transition events at a low nuclear-cytoplasmic ratio
Source: PLoS Biol. 2020 Oct 22;18(10):e3000891. doi: 10.1371/journal.pbio.3000891 (PMC7608951; doi:10.1371/journal.pbio.3000891)

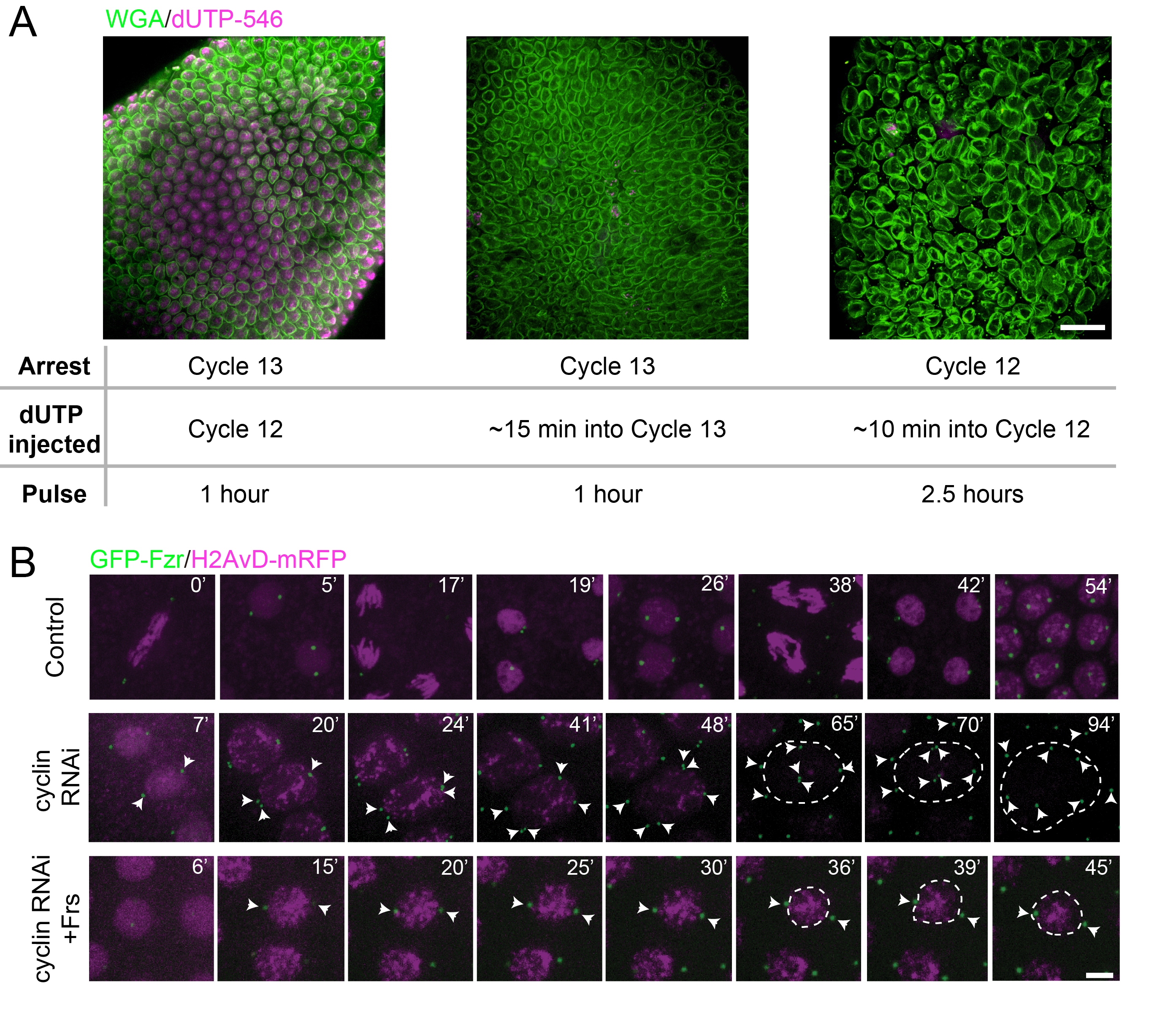

Supplement: S1 Fig — (A) No DNA re-replication after cell cycle arrests. Almost no deoxynucleotide incorporation (purple) is observed when dUTP-546 is injected approximately 10 minutes (cycle 12) or 15 minutes (cycle 13) after entry into an arrested cell cycle. Bar: 10 μm. (B) Arresting the centrosome cycle by cyclin RNAi and Frs protein. When nuclear cycles are arrested by cyclin RNAi, centrosome cycles continue, resulting in about eight centrosomes per nucleus (middle panel, marked by arrows). When embryos are injected with cyclin RNAi and Frs protein, both centrosome and nuclear cycles are stopped, and the centrosome to nucleus ratio remains as 2 (top and bottom panels, marked by arrows). Numbers at the top-right corner in each image are elapsed time (minutes) from the completion of mitosis 10 (top and middle panels) or mitosis 11 (bottom panel). Bar: 5 μm. Frs, Frühstart; RNAi, RNA interference. (TIF) [file pbio.3000891.s001.tif]

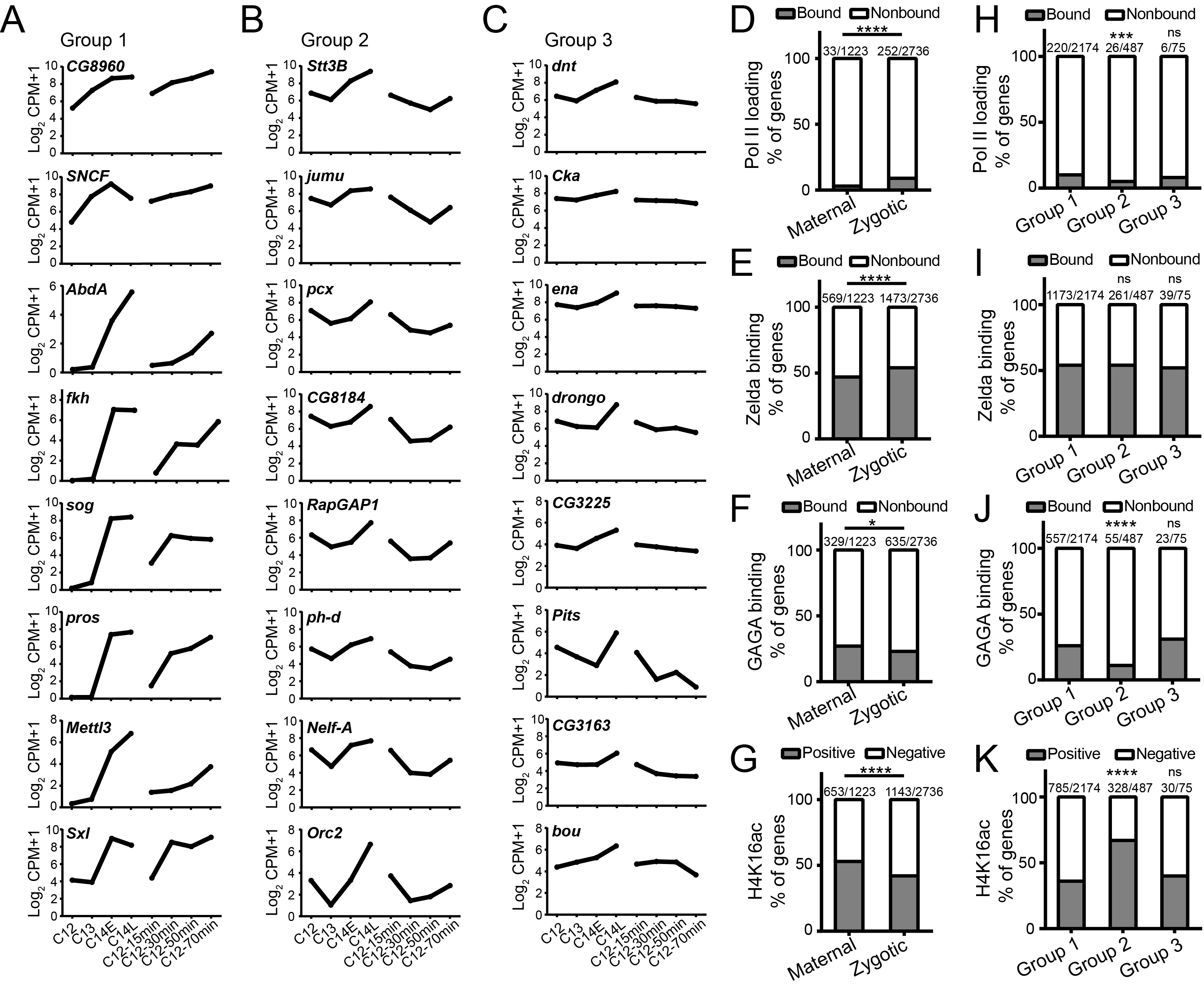

Supplement: S2 Fig — (A-C) Line plots of the Log2 CPM+1 for representative genes from group 1 (A), group 2 (B), and group 3 (C). Related to Fig 3D. (D-G) Differences in RNA Pol II loading, binding of Zelda and GAGA factors, and H4K16ac histone modification between the maternal and zygotic genes analyzed in Fig 3. The zygotic gene list contains more genes that are positive for RNA Pol II loading and Zelda binding (Fisher’s test, p < 0.0001), whereas the maternal gene list includes more genes that have GAGA binding and H4K16ac modification (Fisher’s test, p < 0.05 and p < 0.0001, respectively). (H-K) Comparison of RNA Pol II loading, Zelda and GAGA bindings, and H4K16ac modification states among the three groups of zygotic genes. Differences in Pol II loading, GAGA binding, and H4K16ac modification are observed between group 1 and group 2 (Fisher’s test, p < 0.001, p < 0.0001, and p < 0.0001 respectively). Numerical data for panels A-K can be found in the file S1 Data.xlsx. Log2 CPM+1, Log2-transformed count per million; Pol II, RNA polymerase II; ZGA, zygotic genome activation. (TIF) [file pbio.3000891.s002.tif]

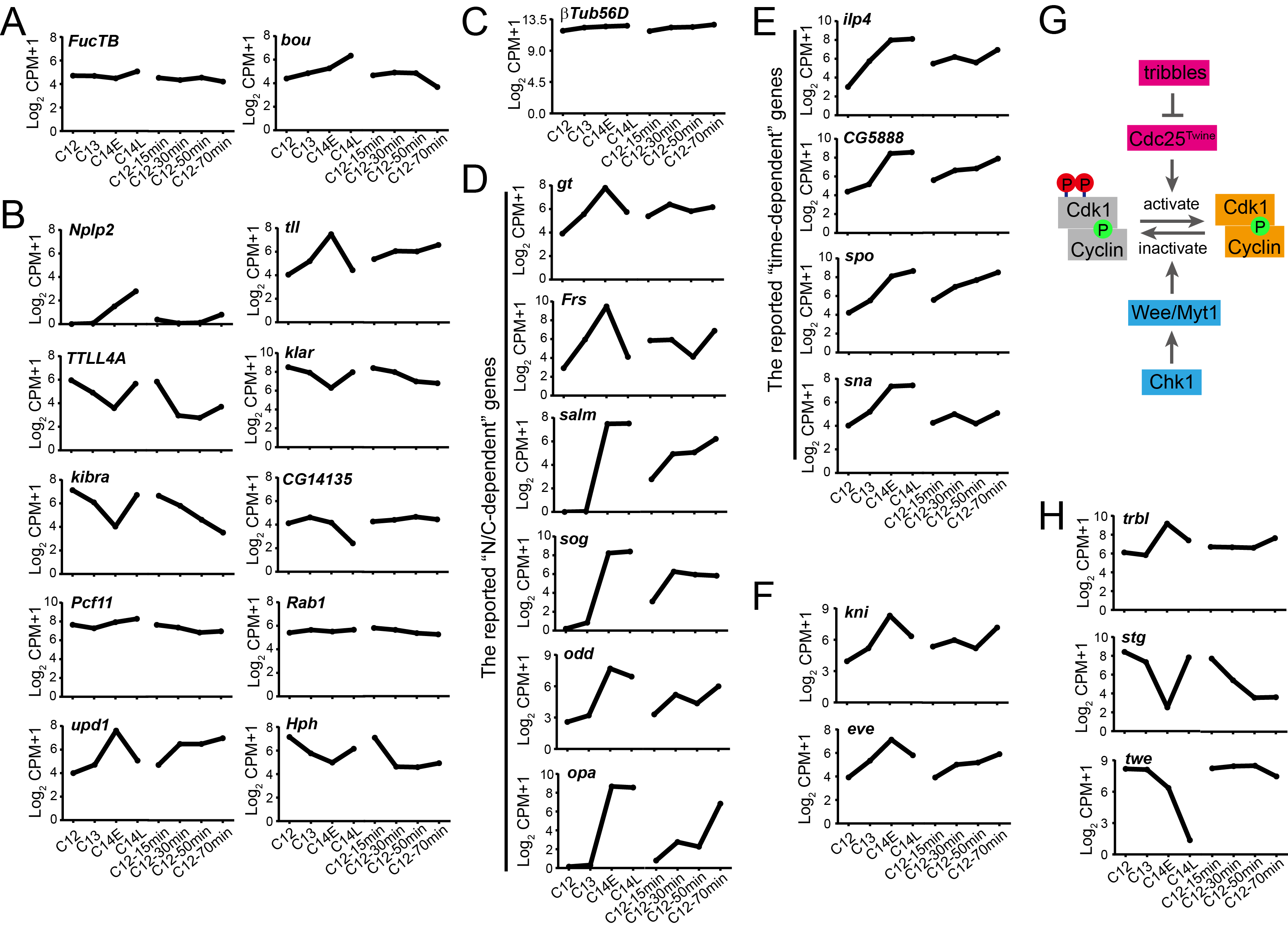

Supplement: S3 Fig — (A) The two “time-dependent” genes that are not increasing their transcripts levels upon cell cycle arrest. (B) Transcriptional profiles for the “N/C-dependent” and “time-dependent” genes that are not included in our analysis (see Fig 4A). (C) The abundance of transcripts of βTub56D are comparable among embryos from different experimental conditions. (D-E) The previously verified “N/C-dependent” genes (gt, Frs, salm, sog, odd, and opa) and “time-dependent” genes (ilp4, CG5888, spo, and sna) increase their mRNA abundance upon cell cycle arrest. (F) kni and eve, the two genes selected for the subsequent nascent transcript imaging experiments, show increased mRNA abundance when the cell cycle is arrested. (G) Developmental control of cyclin/Cdk1. The inhibitory phosphorylation of Cdk1 is added by Wee and Myt1 kinases and removed by Cdc25. There are two Drosophila Cdc25, Cdc25String, and Cdc25Twine. The zygotic transcription of trbl triggers the destruction of Cdc25Twine at the MBT, which results in inactivation of cyclin/Cdk1 and hence cell cycle slowing. (H) The transcriptional profiles of key regulators of the cell cycle in development, trbl, stg, and twe in control and cell cycle–arrested embryos. Numerical data for panels A-F and H can be found in the file S1 Data.xlsx. eve, even skipped; Frs, Frühstart; kni, knirps; MBT, mid-blastula transition; N/C, ratio of nuclei to cytoplasm; odd, odd skipped; salm, spalt major; stg, Cdc25String; trbl, tribbles; twe, Cdc25Twine. (TIF) [file pbio.3000891.s003.tif]

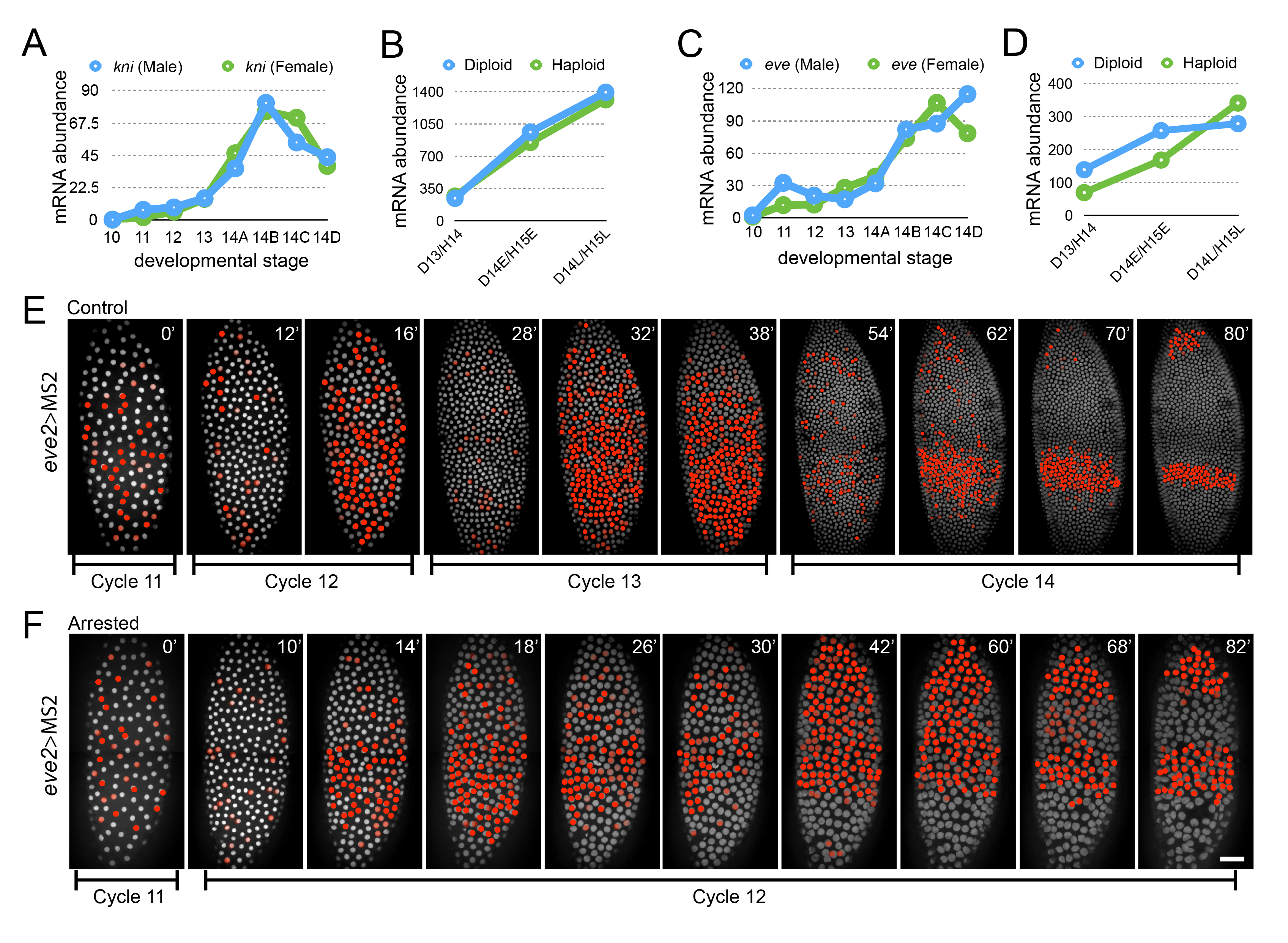

Supplement: S4 Fig — (A) Increase of kni transcript level in cycle 14. The data are adopted from Lott and colleagues [51]. x-Axis is embryonic cell cycles (note that cycle 14 is roughly divided into four stages). (B) The “N/C dependency” of the expression of kni. The expression of kni gene is delayed by one cell cycle in the haploid embryos. The data are adopted from Lu and colleagues [33]. (C) eve mRNA level during early embryonic development. (D) The expression of eve in diploid and haploid embryos. (E) Visualization of eve2 transcriptional activity in control embryos. (F) eve2 transcriptional activity in embryos arrested in cycle 12. Note that a similar but less precise expression pattern is established in the arrested embryos (82’ in panel F versus 80’ in panel E). Nuclei are visualized using mCherry-PCNA. Bar: 50 μm. Numerical data for panels A, B, C, and D can be found in the file S1 Data.xlsx. D13, diploid cycle 13 embryos; eve2, even skipped stripe 2; Frs, Frühstart; H14, haploid cycle 14 embryos; kni, knirps; N/C, ratio of nuclei to cytoplasm; PCNA, proliferating cell nuclear antigen. (TIF) [file pbio.3000891.s004.tif]

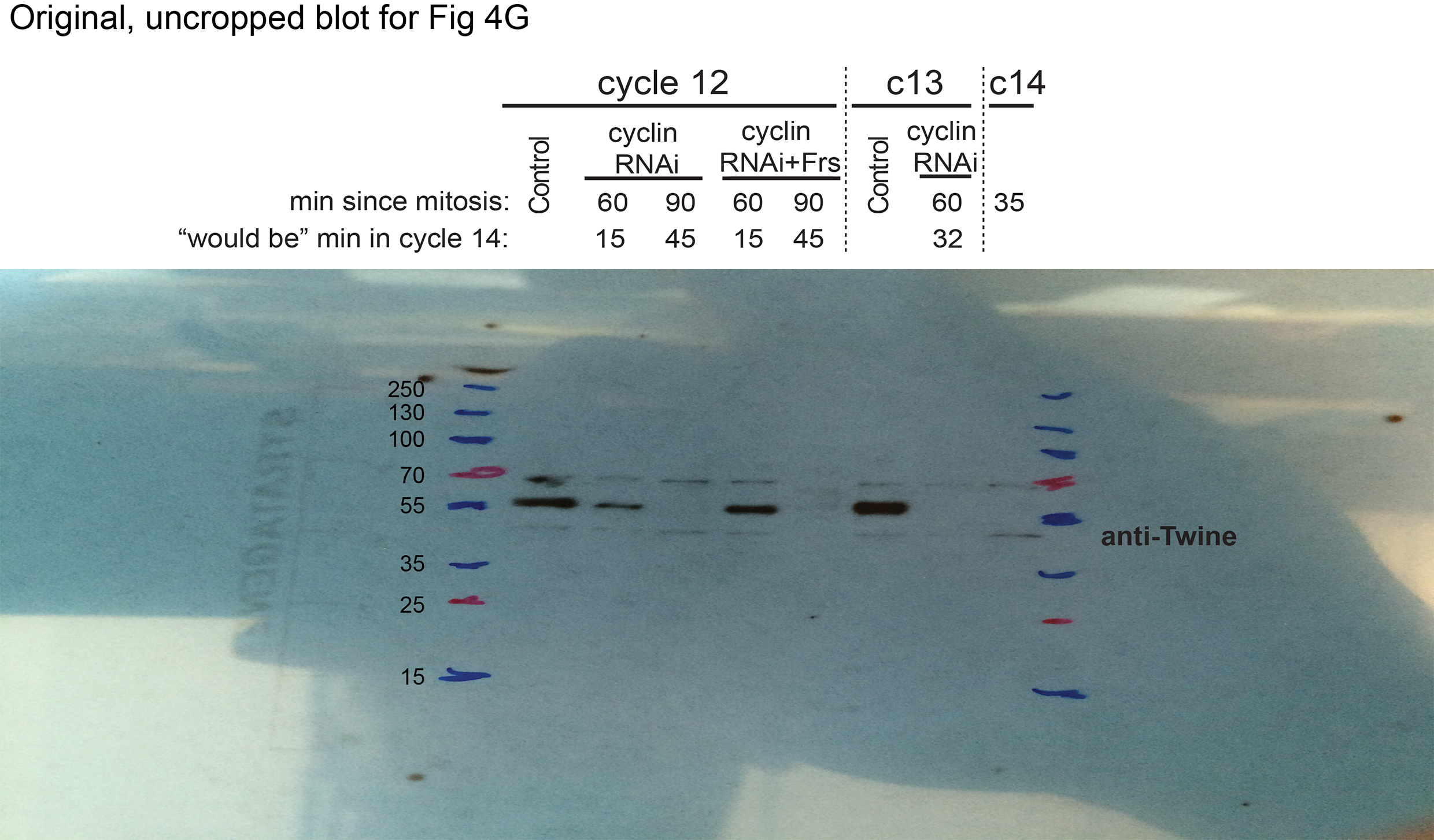

Supplement: S1 Raw Image — (TIF) [file pbio.3000891.s013.tif]
